# Programmable Genome Sensing with Visual, Fluorescence, and Glucometer Readings: Cell-Free *In Vitro* Translation Approach Using RNA Switch-CRISPR

**DOI:** 10.1021/acs.biochem.6c00564

**Published:** 2026-09-16

**Authors:** Mahla Lashkari, Natalie E. Connell, Emmett Hanson, Emmanuel E. Adade, Ram J. Tharu, Mehmet V. Yigit

**Affiliations:** † Department of Chemistry, 1084 University at Albany, State University of New York, 1400 Washington Avenue, Albany, New York 12222, United States; ‡ The RNA Institute, University at Albany, State University of New York, 1400 Washington Avenue, Albany, New York 12222, United States

## Abstract

Rapid and accessible detection of pathogens through diverse detection modalities remains highly desirable. Most current pathogen detection assays rely on a single detection modality. Here, we have used the same RNA switch-CRISPR platform for detection of multiple pathogenic genomes through three different detection modalities. In this study, the RNA switch is repurposed as a reprogrammable platform for genome detection, enabling three orthogonal signal outputs: glucometer-based, fluorescence, and visual. Target pathogen genomes selectively regulate cell-free *in vitro* translation of β-galactosidase through the RNA switch, producing several hundred-fold increases in glucose following lactose hydrolysis while simultaneously generating fluorescence and colorimetric signals. The platform is readily reprogrammed to recognize conserved genomic regions from five distinct pathogens, with specificity dictated by CRISPR–Cas12a-mediated processing of a DNA regulator that controls RNA switch activation and initiates the downstream cell-free translation cascade. Glucometer-based, fluorescence, and paper-based colorimetric outputs each enabled selective detection of *Salmonella typhi*, *Salmonella enterica*, *Listeria monocytogenes*, Shiga toxin-producing *Escherichia coli* (STEC), and *Campylobacter jejuni*. Fluorescence and colorimetric responses were analyzed both qualitatively and quantitatively using image processing and showed strong agreement with glucometer measurements obtained using a handheld glucometer, providing a simple and accessible quantitative readout. The platform was further validated using the complete *Listeria monocytogenes* genome, achieving a detection sensitivity approaching a single genome copy per microliter. Together, this reprogrammable CRISPR-RNA switch platform enables the same detection setup to be programmed for multiple targets and readouts through three different detection modalities, some of which are well suited for resource-limited environments.

## Graphical abstract

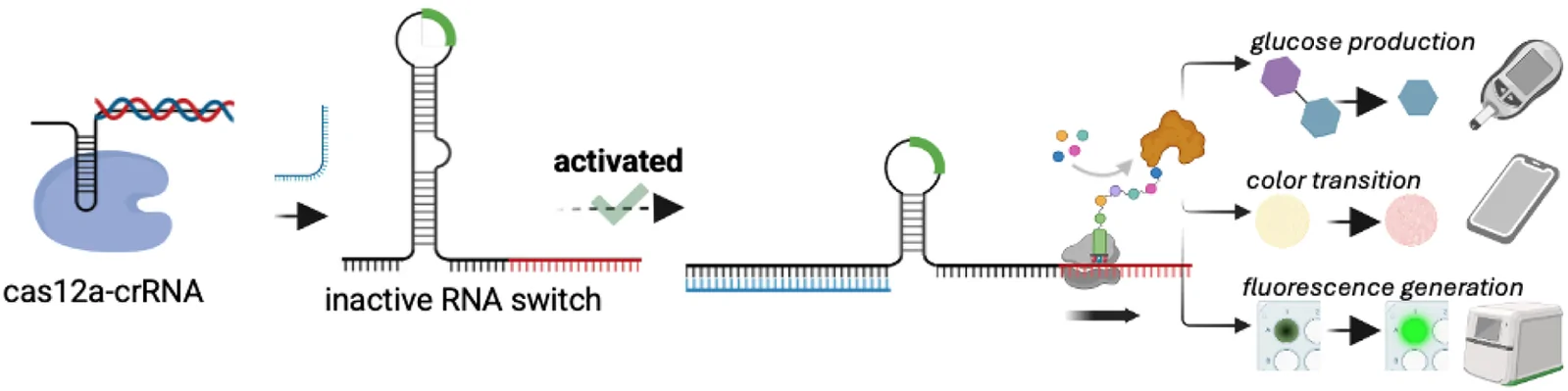

## Introduction

Nature has evolved synergistic biochemical reactions to sustain life. Complex, interconnected processes run continuously within cells with remarkable coordination and precision. The ability to isolate a single metabolic pathway and run it outside the cell can be highly instrumental from an applications standpoint and superior to artificial systems prepared through chemical approaches. Synthetic biology is a field that investigates and mimics desired biological processes in cells, implementing appropriate biochemical modifications to apply them for specific purposes.^1^ Developments in synthetic biology have provided biomolecular tools that can be used in cell-free environments for a variety of vigorous applications.^2–6^

RNA switches are biomolecular tools inspired by nature, in which the ribosome binding site (RBS), start codon, and the gene responsible for translating a protein are embedded within the RNA switch (Figure 1a).^7–9^ In the folded state, the RNA switch masks both the RBS and start codon in a stem-loop structure, making them inaccessible for protein translation. However, once unfolded, the RNA switch cascade enables the activation of protein translation. The translated protein is determined by the gene inserted into the RNA switch. The synthesized protein can be a fluorescent protein, such as GFP,^10^ which can indicate the function of cellular machinery in a cell-free environment, or it can be a different protein (in this case, β-galactosidase) that hydrolyzes substrates and generates quantifiable spectroscopic readouts (Figure 1a).

**Figure 1:**
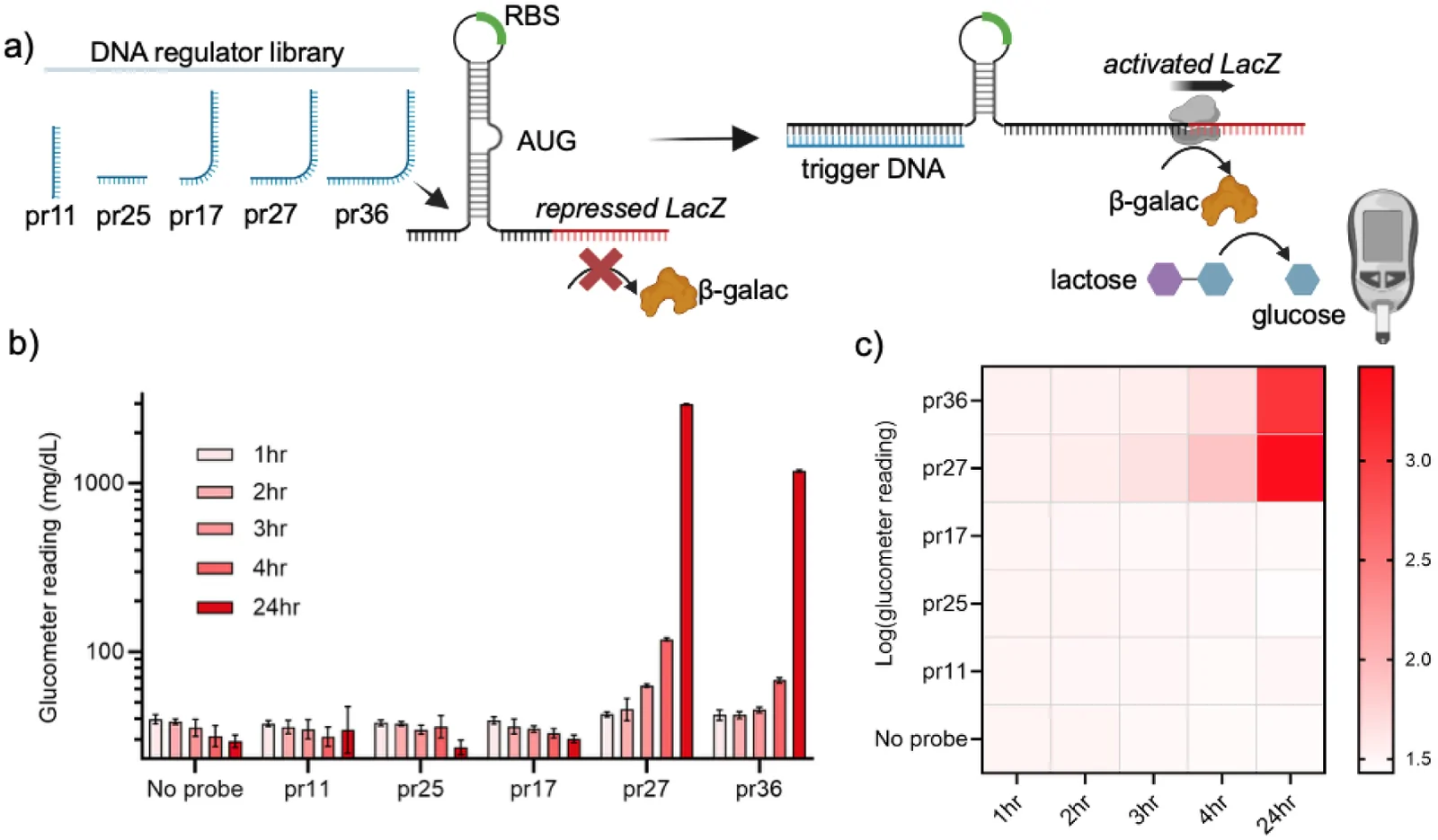
Schematic and experimental workflow for RNA switch activation measured using a glucometer. (a) Schematic illustration of RNA switch activation with five DNA regulators (pr11, pr25, pr17, pr27, and pr36). The activating regulators (pr27 and pr36) bind to the RNA switch, freeing the repressed *LacZ* gene under cell-free conditions, which enables expression of β-galactosidase that catalyzes the hydrolysis of lactose into glucose and galactose; the generated glucose is subsequently quantified using a glucometer. (b) Glucometer measurements obtained with all five DNA regulators at 1, 2, 3, 4, and 24 h suggest that pr27 and pr36 activate the RNA switch, showing a gradual increase in glucometer signal over time. (c) Heatmap representation of the data in (b), highlighting that pr27 and pr36 produce the strongest activation, with the highest glucometer signal observed at 24 h. Experiments were performed in triplicate, and data are presented as mean ± SD.

In this work, we adapted an RNA switch that is activated by sequence-specific nucleic acids to drive the cell-free translation of β-galactosidase, an enzyme that hydrolyzes lactose into glucose and galactose, enabling quantitative glucometer-based detection using a handheld glucometer. Prior work has shown that a sequence-specific RNA oligomer can activate this RNA switch, the activity of which was then verified with a colorimetric indicator for viral detection.^7^ To employ this RNA switch for the intended goals of this report, we investigated and verified that this RNA switch can also be activated by sequence-specific DNA, allowing its activity to be quantified with glucometer readings (Figure 1a). Later, by integrating it with the CRISPR–Cas12a system, the same RNA switch architecture was used for the detection of pathogenic genomes. This strategy allowed us to reprogram the same riboswitch architecture to detect five distinct conserved pathogenic genomic regions using a simple, inexpensive glucometer in addition to fluorescence and visual paper-based detection approaches. Together, these results establish a highly programmable *in vitro* cell-free detection platform capable of targeting any genomic sequence of interest, greatly expanding the potential application space of RNA-based biosensing technologies.

Cas12a technology has transformed nucleic acid-based detection approaches by enabling highly selective recognition of conserved genomic regions.^11–13^ This capability arises from Cas12a’s sequence-specific DNA binding followed by activation of collateral cleavage of nearby single-stranded DNA (ssDNA).^14,15^ When combined with additional biological processes, assay sensitivity can be substantially enhanced, and signal outputs can be modulated.^16^ Moreover, this integration allows biological toolkits originally designed for a single target to be readily adapted for the detection of multiple markers.^17–19^ Here, we demonstrate that pairing Cas12a with an RNA switch encoding the *lacZ* gene enables us to reprogram the system to detect five different bacterial pathogens, with readout achieved through three distinct signal modalities: paper-based visual detection, fluorescence, and glucometer-based measurements.

Bacterial and viral pathogens pose serious risks to human health and the global economy.^20,21^ Early and rapid detection is essential to limit transmission and prevent large-scale outbreaks.^22^ Point-of-care diagnostics are especially valuable because they enable testing outside laboratories and can be performed by minimally trained personnel.^23^ This improves response time and supports effective disease control.^24^ Among foodborne pathogens, *Salmonella* species are associated with significant economic and health burdens.^25^ *Campylobacter jejuni* is a leading cause of gastroenteritis and can lead to Guillain-Barré syndrome.^26^ Shiga toxin-producing *Escherichia coli* (STEC), particularly O157:H7, can cause hemolytic uremic syndrome and acute kidney failure, especially in children and the elderly.^27^ *Listeria monocytogenes* threatens pregnant women and immunocompromised individuals and may cause meningitis, septicemia, or fetal loss.^28^

Given these risks, the development of a diagnostic platform capable of detecting multiple bacterial contaminants offers clear advantages for healthcare systems and public health. Although several glucometer-based biosensing strategies have been reported,^29,30^ many rely on novel magnetic bead-mediated separation steps for enzyme-conjugated systems^31^ or require the design of target-specific RNA switches for each individual target.^32^ In contrast, the system described here eliminates the need for separation steps and enables reprogramming of a single RNA switch architecture toward diverse genomic targets. Furthermore, it integrates three independent signal output modalities within the same platform, enhancing versatility and practical applicability.

RNA-based switches have been explored for many years as programmable tools for controlling gene expression and biological sensing. Early studies from the Smolke group demonstrated that aptamer-based sensing elements could be incorporated to generate modular RNA devices capable of responding to specific inputs and regulating downstream gene expression.^33,34^ More recent studies have expanded RNA-switch platforms for applications including pathogen detection, cell-free sensing, and quantitative point-of-care readouts.^8,35,36^ In many of these systems, however, target recognition is encoded directly within the RNA sensing or switching element, such that adaptation to a new nucleic acid target may require redesign or re-engineering of the RNA component. In the present work, we instead employ CRISPR–Cas12a as a programmable target recognition layer that regulates a downstream RNA switch through processing of a DNA regulator. This approach separates nucleic acid target recognition from the RNA switch, enabling the same RNA switch to be redirected toward different targets while maintaining multiple downstream readout modalities. By integrating programmable CRISPR–Cas12a recognition with an RNA-based switching mechanism, our approach extends previous RNA-switch architectures and provides a modular strategy for coupling target-specific nucleic acid recognition to RNA-switch outputs. The advantages and limitations of the RNA-switch-CRISPR platform relative to conventional CRISPR-based and RNA-switch systems are summarized in Table 1.

**Table 1:** Comparison of RNA Switch-CRISPR Detection Platform with RNA Switch- and CRISPR- Alone Systems

Technology	Pros and cons	Example of application
RNA switch-CRISPR	**Pros:** Highly sequence-specific recognition with programmable target detection. Can be coupled to multiple accessible readout modalities (glucometer-based, fluorescence and colorimetric). Improved sensitivity through multiple intrinsic amplification steps. Quantitative and semi-quantitative readouts.	Selective detection of pathogen genomes in a cell-free *in vitro* system (e.g., This study and Kachwala et al.^11^).
	**Cons:** Requires prior sequence information for guide RNA design.	
RNA switch (paper-based)	**Pros:** Sequence specific genome recognition, inexpensive, portable and suitable for point of care applications. Semi-quantitative.	Paper-based analysis of microbiota and host markers (e.g., Takahashi et al.^2^).
	**Cons:** Requires redesign of toehold switch for each new target and generally lacked multiplex target discrimination in a single reaction. Requires prior sequence information	
CRISPR-based fluorescence detection	**Pros:** Rapid, highly sensitive fluorescence-based nucleic acid detection	Fluorescence-based CRISPR diagnostic platform (e.g., Chen et al.,^14^ Hanson et al.^12,37^ ).
	**Cons:** Detection is generally limited to fluorescence readout and often requires specialized fluorescence instrumentation. Requires prior sequence information.	

## Materials and Methods

### Materials

The toehold switch (ZIKV_Sensor_27B_LacZ; plasmid #75006) was purchased from Addgene (Watertown, MA, USA). All DNA and crRNA constructs were obtained from Integrated DNA Technologies, Inc. (Coralville, IA, USA) (see Table S1). PURExpress *In Vitro* Protein Synthesis Kit (catalog #E6800L), Monarch PCR & DNA Cleanup Kit (5 μg; catalog #T1030S), EnGen Lba Cas12a (Cas12a) (catalog #M0653), and NEBuffer 2.1 10X reaction buffer (catalog #B6002S) were purchased from New England Biolabs (Ipswich, MA, USA). The RPA kit (catalog #TABAS03KIT) was obtained from TwistDx Limited (Cambridge, UK). The *Listeria monocytogenes* EGD-e genome (catalog #BAA-679D-5, batch #70071410) was purchased from the American Type Culture Collection (ATCC; Manassas, VA, USA). Bovine serum albumin (BSA) (catalog #9048-46-8) and magnesium chloride hexahydrate (catalog #7791-18-6) were purchased from Amresco (Solon, OH, USA). LB Broth, Miller molecular genetics powder (catalog #245960-2 kg) and RNase-free distilled water were purchased from Thermo Fisher Scientific (Waltham, MA, USA). Kanamycin sulfate (catalog #75856-684, batch # 22E235311), LB agar plate with 50 μg/mL kanamycin (catalog #100216-588), ZymoPURE II Plasmid Maxiprep Kit (catalog #77001-470), Integra Miltex Disposable Punch Biopsy, 2 mm (catalog #21909-132), 0.2 μm syringe filter (catalog #28145-487), 96-well half-area plate (catalog #29444-316) and 384-well plate (catalog #89089-862) was purchased from VWR International (Secaucus, NJ, USA). Chlorophenol Red-β-D-galactopyranoside i.e., CPRG (catalog #59767-100MG-F), fluorescein di-β-D-Galactopyranoside (FDG), i.e., FDG (catalog #17796LHV-1MG), D-lactose monohydrate (catalog #61339-25G), Whatman filter paper (catalog #WHA1442042), Protector RNase Inhibitor (catalog #3335399001), and all other chemicals were purchased from Sigma-Aldrich (St. Louis, MO, USA). Phosphate-buffered saline, pH 7.4 (1X, 10 mM), was purchased from Thermo Fisher Scientific Inc. (Waltham, MA, USA).

### Equipment

Nucleic acid concentration was measured using an Agilent Technologies Cary 60 UV–vis spectrophotometer. An Agilent BioTek Synergy H1 Multimode Plate Reader was used for microplate fluorescence and absorbance measurements. All incubation steps were performed using a Bio-Rad T100 Thermal Cycler. Fluorescence imaging was conducted with a Bio-Rad ChemiDoc MP Imaging System. Color change images were captured using an Eclipse TS100 microscope. An Eppendorf Centrifuge 5424 was employed for centrifugation during RPA amplicon cleanup. Glucose level was measured using a Metene TD-4116 Blood Glucose Monitor Kit. Figures and graphical illustrations were created using BioRender (biorender.com) and GraphPad Prism (graphpad.com).

### RNA Switch Isolation

The RNA switch plasmid obtained from Addgene (plasmid #75006) was maintained as glycerol stocks for long-term storage at −80 °C. To isolate pure bacterial single colonies, the glycerol stock was then streaked onto LB agar plates containing 50 μg/mL kanamycin and incubated at 37 °C overnight. A single colony of bacteria was inoculated in 5 mL sterile LB broth (with 50 μg/mL kanamycin) as a starter culture, and then it was kept in a shaking incubator at 37 °C and 220 rpm for 24 h. Thereafter, 1/250th volume of starter culture was added to large-volume LB broth containing 50 μg/mL kanamycin to prepare the main culture, e.g., 1 mL of starter culture was added to 250 mL of LB broth and incubated in the shaking incubator at 37 °C and 200 rpm for at least 20 h. The plasmid was purified using the ZymoPURE Plasmid Purification Kit following the manufacturer’s protocols optimized for low-copy plasmids. Subsequently, the purified DNA was concentrated using the Monarch Cleanup Kit (New England Biolabs). The RNA switch concentration was determined via absorbance measurements at 260 nm with the molar extinction coefficient 61,243,100 M^–1^ cm^–1^ using Agilent Technologies Cary 60 UV–vis spectrophotometer. The molar extinction coefficient was calculated by inputting the plasmid DNA sequence into the IDT OligoAnalyzer tool. The main stock of the RNA switch plasmid was diluted into separate 50 nM stocks and stored at −20 °C.

### Primers, Truncated Fragments, and crRNA Design

To activate the RNA switch, five DNA probes were designed using the Integrated DNA Technologies (IDT) design platform with different lengths (36, 27, 25, 17, and 11 nucleotides). The sequences of probes pr36, pr27, pr25, pr17, and pr11 are listed in Table. S1. To identify targetable regions in *Listeria monocytogenes* (Listeria), Shiga toxin-producing *E. coli* (STEC), *Campylobacter jejuni* (*C. jejuni*), *Salmonella typhi* (*S. typhi*), *Salmonella enterica* (*S. enterica*) genomes, conserved genomic regions containing a canonical TTTN protospacer adjacent motif (PAM) and relatively high GC content were selected through sequence analysis using the NCBI Nucleotide BLAST tool against the core nucleotide database (core_nt). Based on these sequences, the corresponding crRNAs were designed via the Integrated DNA Technologies (IDT) website. For Listeria, the *hly* gene was identified as containing the target conserved region through sequence analysis using the NCBI Nucleotide BLAST tool. After confirming a conserved region within the *hly* locus in *Listeria monocytogenes* EGD-e (AL591824), RPA primer pairs (Table S1) were designed using SnapGene software (Insightful Science, snapgene.com) based on GC content, target site compatibility, and adherence to the manufacturer’s RPA kit guidelines. All nucleotide sequences used in this study are provided in Table S1. The RPA primers used in this study were selected from primer sets evaluated in our previous study. The combination of forward primer 2 (F2) and reverse primer 4 (R4) was used for the RPA studies.

### Oligonucleotide Preparation

All double-stranded DNA (dsDNA) stocks were initially dissolved in RNase-and DNase-free deionized (RDI) water to prepare 0.1 mM stock solutions. The 100 μM oligonucleotides were further diluted to form 10 μM stocks in 1× PBS. All primers were dissolved in RDI water to a final concentration of 10 μM. The corresponding crRNA(s) were prepared in 1× Tris-EDTA (TE) buffer (pH 7.9). According to the ATCC certificate of analysis, *Listeria monocytogenes* EGD-e genomic DNA was dissolved in 250 μL of RDI water to prepare a stock solution with a concentration of 1.28 × 10^7^ copies/μL. All oligonucleotide’s concentrations of all oligonucleotides were confirmed by optical density at 260 nm using an Agilent Technologies Cary 60 UV–Vis spectrophotometer.

### Evaluating Toehold Activation Using Different Probes

Stock solutions of the substrates for β-galactosidase were prepared as follows: lactose (380 mg/mL), CPRG (20 mg/mL), and FDG (1.5 M) were dissolved in phosphate-buffered saline (PBS), RDI water, and methanol, respectively.

### Color Transition on Filter Paper Substrates

To prepare BSA-treated paper, the Whatman filter paper was first treated by submerging it in a 5% BSA solution (prepared in 1× PBS buffer) on a shaker (30 rpm) for 24 h. After 24 h, the paper was rinsed twice with RDI water for 10 min. Once completely dry, - mm paper discs were punched using a biopsy punch and placed in a dark 384-well assay plate. According to the PURExpress *In Vitro* Protein Synthesis Kit, a protein master mix was prepared containing: 1.08 μL of solution A, 0.81 μL of solution B, 0.05 μL of 40 U/μL RNase inhibitor, 1.5 μL of 50 nM isolated toehold switch plasmid, and 0.2 μL of 20 mg/mL CPRG. Next, 3.64 μL of the prepared protein mix was added to a microtube (0.2 mL) for each reaction. Subsequently, 1 μL of each probe (pr11, pr25, pr17, pr27, pr36) from a 232 nM stock was added individually to the corresponding microtubes to reach a final concentration of 50 nM. Finally, the total volume of 4.64 μL was transferred onto the paper discs in the plate. All preparation steps were performed on ice. The plate was placed in a preheated Agilent BioTek Synergy H1 Multimode spectrophotometric microplate reader (37 °C), and absorbance at 570 nm was measured over 2 h at 37 °C. Post-incubation, images were captured at 2× magnification using an Eclipse TS100 microscope equipped with a digital camera. The experiments were repeated to monitor image contrast using different concentrations of CPRG (1× = 0.862 mg/mL, 2×, 3×, 4×, and 5×) with pr27 (50 nM in each reaction). To determine the concentration dependence of RNA switch activation on the DNA regulator pr27, various concentrations (2 nM, 10 nM, 50 nM, 100 nM) of pr27 were tested using 1× CPRG.

### Fluorescence Measurements in the Liquid Phase

Fluorescence-based measurements were performed in the liquid phase. For cell-free protein expression, all reagent volumes were maintained according to the aforementioned protocol with CPRG. A protein master mix was prepared containing: 1.08 μL of solution A, 0.81 μL of solution B, 0.05 μL of 40 U/μL RNase inhibitor, 1.5 μL of 50 nM isolated RNA switch plasmid, and 0.2 μL of 15 mM FDG. Next, 3.64 μL of the prepared protein mix was added to a 0.2 mL microtube for each reaction. 1 μL of probe (pr36, pr27, pr25, pr17, pr11 from a 232 nM stock) was added to the 3.64 μL protein mix in the microtube separately for each reaction. Next, the final reaction volume (4.64 μL) was transferred to a 384-well plate for each reaction. All preparation steps were performed on ice. Subsequently, fluorescence was measured at excitation 485 nm and emission 520 nm for 2 h at 37 °C. Following completion of the reaction, imaging was performed by placing 2 mm punched Whatman filter paper discs into the wells to enhance image contrast. Images were obtained using the Bio-Rad ChemiDoc MP Imaging System. The experiments were repeated to monitor fluorescence contrast using various concentrations of FDG (1× = 0.065 mM, 2×, 5×, 10×, 20×) with pr27 (50 nM in each reaction). To assess the concentration-dependence of RNA switch activation on the DNA regulator (pr27), various concentrations (2 nM, 10 nM, 50 nM, 100 nM) of pr27 were tested using 10× (0.65 mM) FDG.

### Monitoring the Glucose Level through Lactose Hydrolysis

For lactose hydrolysis through expressed β-galactosidase upon RNA switch activation, 380 mg/mL lactose was prepared in phosphate-buffered saline (1×, pH 7.4) (PBS) and stirred at 40–50 °C until completely dissolved completely. Subsequently, the solution was filtered through a 0.2 μm membrane filter. The protein master mix, comprising 2.16 μL of solution A, 1.62 μL of solution B, 0.1 μL of 40 U/μL RNase inhibitor, 3 μL of 50 nM isolated RNA switch plasmid, and 1 μL of 380 mg/mL lactose, was prepared in a microtube on ice. Following this, 2 μL of DNA regulators (pr36, pr27, pr25, pr17, pr11; 247 nM stock) were added to a final concentration of 50 nM. The microtubes were incubated at 37 °C for 1, 2, 4, 24, 48, and 72 h in a Bio-Rad T100 Thermal Cycler. At each time point, reactions were mixed evenly using a pipette, and the glucose level was measured with a Metene TD-4116 Blood Glucose Monitor Kit using 0.7 μL of sample. The corresponding data were recorded in an Excel spreadsheet and plotted with GraphPad Prism software on a logarithmic scale. Some reactions produced overflow data due to high glucose readings; these samples were diluted tenfold, and the final glucose reading (mg/dL) was back-calculated by multiplying the readings by 10.

### CRISPR–Cas12a with the Toehold Reaction

All CRISPR–Cas12a reactions were prepared using NEBuffer r2.1 (1×) as the Cas buffer throughout the experiment. Cas12a (100 μM) and the target crRNA were combined at a 1:1.2 molar ratio in a final volume of 50 μL and incubated at 37 °C for 30 min to allow formation of the Cas12a–crRNA complex. Following complex formation, 2 μL of the complex was combined with the Cas buffer and Listeria dsDNA at different concentrations (1 nM, 200 nM, 40 pM, 8 pM, and 1 pM) in a final volume of 16.2 μL, and incubated for an additional 30 min at 37 °C. Then, 3.8 μL of probe pr27 (1 μM) was added to achieve a final volume of 20 μL (190 nM), and the reaction was incubated at 37 °C for 2 h, followed by a 15 min deactivation of the Cas12a enzyme at 65 °C. This CRISPR reaction was used for the subsequent experiments.

### Color Transition on Filter Paper Substrates with RNA Switch-CRISPR

The Cas12a–crRNA complex was prepared using Cas12a and crRNA at a 1:1.2 molar ratio according to the aforementioned procedure. 1.5 μL of the Cas12a reaction containing pr27 (190 nM) was mixed with the protein master mix containing 1.08 μL of solution A, 0.81 μL of solution B, 0.05 μL of 40 U/μL RNase inhibitor, 1.5 μL of 50 nM isolated RNA switch plasmid, and 0.2 μL of freshly prepared CPRG (20 mg/mL). The components were combined in a 0.2 mL microtube. The final reaction volume was 5.14 μL, containing 3.64 μL of the protein master mix and 1.5 μL of the Cas12a reaction. The RNA switch resulted in either a color transition (yellow to red/purple) or none in response to activation of the Cas12a–crRNA reaction. A final volume of 5.14 μL was dispensed individually into the wells of the 384-well plate containing the paper discs. To avoid premature initiation of the reaction, all experimental steps were conducted on ice. The plate was then transferred to the microplate reader set to 37 °C, and absorbance at 570 nm was monitored for 2 h at 37 °C. After incubation, images were acquired at 2× magnification using an Eclipse TS100 microscope (Nikon) equipped with a camera.

### Fluorescence Measurements in the Liquid Phase with RNA Switch-CRISPR

A 1.5 μL aliquot from each Cas12a reaction (various dsDNA target concentrations) was added to the protein master mix, consisting of 1.08 μL of solution A, 0.81 μL of solution B, 0.05 μL of 40 U/μL RNase inhibitor, 1.5 μL of 50 nM isolated RNA switch plasmid, and 0.2 μL of 15 mM freshly prepared FDG. The mixture was prepared in a 0.2 mL microtube. A 5.14 μL aliquot of the resulting reaction mixture was added to each well. Preparation steps were performed on ice. Samples were placed in a microplate reader preheated to 37 °C to collect fluorescence readings. Fluorescence signals were recorded for 2 h at 37 °C with excitation at 485 nm and emission at 520 nm. After completion of the reaction, 2 mm-diameter punched discs of Whatman filter paper were placed in the wells to enhance image contrast. Images were then collected using a Bio-Rad ChemiDoc MP Imaging System.

### Monitoring the Glucose Level with RNA Switch-CRISPR

For CRISPR experiments, 3 μL of each Cas12a reaction, with various dsDNA target concentrations as described above and containing pr27 (190 nM), was mixed with a protein master mix containing 2.16 μL of solution A, 1.62 μL of solution B, 0.1 μL of 40 U/μL RNase inhibitor, 3 μL of 50 nM isolated RNA switch plasmid, and 1 μL of freshly prepared lactose (380 mg/mL), and the mixture was combined in a 0.2 mL microtube. As a result, a total volume of 7.88 μL of the protein master mix was mixed with 3 μL of the Cas12a reaction, yielding a final reaction volume of 10.88 μL per microtube. Reactions were incubated in a Bio-Rad T100 Thermal Cycler at 37 °C, and measurements were recorded at 1, 2, 4, and 24 h using a Metene TD-4116 Blood Glucose Monitor Kit, with a 0.7 μL sample used per measurement.

### Specificity Mapping and Multitarget Detection

The grid box, consisting of a 5×5 matrix configuration, allowed parallel RNA switch-CRISPR detection reactions. Each row represents specific crRNA(s), while each column corresponds to a distinct target sequence. This orthogonal arrangement allowed simultaneous evaluation of all 25 possible combinations. CRISPR–Cas12a reactions were prepared for each row according to the aforementioned procedure (2 μL of Cas12a–crRNA complex with 3.8 μL of 1 μM pr27) in separate microtubes, each containing 5 nM of a corresponding genomic target. The samples were then incubated for 2 h at 37 °C, followed by a 15-min deactivation of the Cas12a enzyme at 65 °C. Next, each CRISPR–Cas12a reaction was combined with the protein master mix of the toehold switch reaction according to the aforementioned procedure in each corresponding experiment for glucometer, fluorescence, and color-transition-based detection. Following the combination of CRISPR–Cas12a with the RNA switch reactions, the mixtures were incubated in a microplate reader for 2 h at 37 °C, and the corresponding measurements were recorded. For obtaining images of color transitions on paper clips, each well was imaged at 2× magnification using an Eclipse TS100 microscope with a camera attachment. Fluorescent images were captured after the completion of the microplate readout following the addition of 2 mm punched paper discs to the wells to enhance contrast for fluorescence imaging using the Bio-Rad ChemiDoc MP Imaging System. All images were recorded at the same moment using the same parameters to avoid misrepresentation. The glucose level of each reaction was measured using the Metene TD-4116 Blood Glucose Monitor Kit at 24, 48, and 72 h post-incubation at 37 °C in the thermocycler and recorded in an Excel spreadsheet for data analysis. Reaction volumes, incubation conditions, and reagent concentrations were kept constant across all grid compartments to ensure comparability.

### RPA Primer Screening for *Listeria monocytogenes* EGD-e

To identify the optimal combination of RPA primers, four forward and four reverse primers were selected and tested against each other for Cas12a activation using an F-Q probe, (Table S1 and Figure S7). Following the TwistAmp Basic Kit Quick Guide, pellets were rehydrated with 29.5 μL of primer-free rehydration buffer. For every combination of forward and reverse primers, totaling 32 tests (16 genome-positive and 16 genome-negative), 2.4 μL of 10 μM selected forward primer and 2.4 μL of 10 μM selected reverse primer were then added. Next, 1 μL of full *Listeria monocytogenes* EGD-e genome (10^3^ copies/μL) was added to each sample, with 1 μL of RDI buffer used for genome-negative samples. To activate the RPA reaction, 2.5 μL of 280 nM magnesium acetate (MgOAc) was added, followed by mixing with the same pipette. All samples were incubated at 39 °C for 20 min. After the short incubation period, 8 μL of each RPA solution were added to separate wells in a 96-well plate along with 8 μL of 250 nM F-Q probe, 19 μL of 1× NEBuffer 2.1, and 5 μL of Cas12a complex (125 nM Cas12a and 150 nM Listeria crRNA). The plate was then placed in a preheated plate reader (37 °C), and fluorescence was measured with excitation at 485 nm and emission at 528 nm every minute for 60 min total.

### Preparation of Listeria Amplicons from the Listeria Genome Using RPA

The *Listeria monocytogenes* EGD-e genome was purchased from ATCC (American Type Culture Collection), and according to the ATCC product sheet, the original stock was supplied as 5 μg of dried genomic DNA (total length of the DNA is 2.9 Mb). The genome sample was dissolved in 250 μL of RNase-and DNase-free deionized (RDI) water to prepare a 20 ng/μL stock and was stored at 4 °C overnight, according to the standard procedure provided by ATCC. According to standard double-stranded DNA mass calculations, 1 ng of this DNA contains roughly 3.10 × 10^5^ genome copies.^38,39^ Thus, the reconstituted stock contained approximately 6.20 × 10^6^ genome copies/μL. Two low-copy-number working stocks, 10^3^ copies/μL (S1) and 10^4^ copies/μL (S2), were subsequently prepared from the original stock by dilution. Recombinase Polymerase Amplification (RPA) was performed using the TwistAmp Basic Kit, following the manufacturer’s Quick Guide for solid-phase RPA.

For genome detection, each RPA reaction contained 29.5 μL Primer-Free Rehydration Buffer, 2.4 μL each of 10 μM forward (F2) and reverse (R4) primers, 12.2 μL RDI water, and 1 μL of S1 or S2. After mixing, 2.5 μL of 280 mM magnesium acetate (MgOAc) was added to the resulting mixture, followed by brief vortexing. Three parallel reactions were prepared: two with positive controls corresponding to S1 or S2 with genomic DNA (10^3^ or 10^4^ copies/μL), and one with 1 μL of RDI water as a negative control. Reactions were incubated for 20 min at 39 °C. Subsequently, the RPA products (∼50 μL) were purified using the Monarch Spin PCR & DNA Cleanup Kit, following the oligonucleotide cleanup protocol. For purification, each reaction was transferred to an individual spin column (placed in a collection tube) with 100 μL binding buffer and 300 μL isopropanol, mixed by pipetting, and then centrifuged at 13,000 rpm for 1 min. The flow-through was discarded, and the columns were washed twice with 500 μL wash buffer by centrifugation at 13,000 rpm for 1 min. Finally, 20 μL of elution buffer was added to each column, incubated at room temperature for 5 min, and then centrifuged at 13,000 rpm for 1 min. The final 20 μL eluted products (RPA-P1 and RPA-P2), containing approximately 10^3^ and 10^4^ amplicons in total and corresponding to concentrations of 50 and 500 genome copies/μL in the RPA reaction, were collected and used as the target templates for subsequent assays.

### Detection of the Listeria Genome

All detection assays using the RPA-generated amplicons (RPA-P1 = 50 genome copies/μL and RPA-P2 = 500 genome copies/μL) were prepared according to the procedures mentioned above, except that the RPA amplicons were used as the target. The assays were tested using 4 μL and 8 μL of both RPA-P1 and RPA-P2 in detection reactions with a final volume of 40 μL. Based on the initial template input, these volumes correspond to 200 and 400 genome-copy equivalents for RPA-P1 and 2000 and 4000 genome-copy equivalents for RPA-P2 per 40 μL detection reaction, respectively, or 5, 10, 50, and 100 genome-copy equivalents/μL.

To evaluate sensitivity with the whole genome, all colorimetric, fluorescent, and glucometer-based assays were performed with 1 μL, 2 μL, 4 μL, and 8 μL of the RPA-P1 sample. Thus, 1, 2, 4, and 8 μL of RPA-P1 were used in detection reactions with a final volume of 40 μL. Based on the initial template input, these volumes correspond to 50, 100, 200, and 400 genome-copy equivalents per 40 μL detection reaction, respectively, or 1.25, 2.5, 5, and 10 genome-copy equivalents/μL. The positive controls in the assays were performed using synthetic targets at a 5 nM concentration, whereas negative controls were performed using RPA products without Listeria genomic DNA (negative control in the RPA assay). Specificity was evaluated using four different crRNAs (against *STEC*, *C. jejuni*, *S. typhi*, and *S. enterica*)) that were not designed for Listeria.

### Colorimetric Analysis of CPRG Assay Images

RGB images of CPRG assay paperclips were acquired using a Nikon Eclipse TS100 microscope equipped with a digital camera under the same acquisition settings. Individual images were compiled into a grid (5 × 6 or 5 × 5). The compiled grid images were imported into MATLAB and converted from RGB to CIE L*a*b* color space using a standard color space transformation function.^40^ This color space separates luminance (L*) from chromatic components (a*, b*), thereby reducing noise introduced by variations in brightness and enabling more robust color-based quantification. Analysis was performed on the a* channel, which represents the red-green color axis and provides a sensitive measure of chromogenic changes associated with CPRG substrate conversion. Although the b* channel was also evaluated, it contributed limited additional information in this dataset and was therefore excluded from further analysis.

The a* channels were extracted from each image, and a clear contrast corresponding to a red chromogenic shift was observed in the processed images (Figures S4 and S6). Circular assay regions (∼2 mm in diameter, corresponding to ∼17,000 pixels per region) were identified using a built-in MATLAB circle-detection function with empirically optimized parameters. Subsequent analyses were restricted to pixels within these detected circular regions. Each circular region was subdivided into square grid blocks of fixed size, defined relative to image resolution to ensure adequate pixel sampling (e.g., 10 × 10 or 15 × 15 pixels, depending on the image resolution). Only blocks with at least 95% area overlap within the detected circle were retained to minimize edge and background effects. Pixel-based quantitative analysis was performed on the a* channel, with the a* coordinate defined as a* = *C*_*ab*_ × cos­(h_*ab*_), where *C*_*ab*_ represents chroma and *h*_*ab*_ represents the hue angle in CIE L*a*b* space, as previously described by Komatsu et al.^40^ For each retained block, the mean a* value was calculated from all included pixels and used as a quantitative measure of the local colorimetric signal. Block-wise mean a* values were visualized as heatmaps to represent spatial color variation in the CPRG signal across the assay surface. All image processing and quantitative analyses were performed in MATLAB version R2021b Update 6 (9.11.0.0.2207237).

The MATLAB code used for image analysis, together with a representative test image provided solely for demonstration and code execution, is available on GitHub at https://github.com/Edem2326/Yigit-lab-CPRG-colorimetric-image-analysis.git. All datasets supporting this analysis, including raw and processed images, are available on Zenodo under 10.5281/zenodo.19556047.

## Statistical Analysis

The experiments were performed in triplicate, and the error bars were calculated using the standard deviation (SD). Data are represented as mean ± SD.

## Results and Discussion

### Activating the RNA Switch with DNA Regulators Using Glucometer, Fluorescence, and Visual Outputs

First, the working principle of the RNA switch was evaluated by using several DNA regulators as triggers that could potentially activate the switch. Prior studies demonstrated that this RNA switch architecture can be activated by a small RNA trigger.^7^ To assess this capability with DNA regulators, five different DNA candidates were tested. Three candidates (pr17, pr27, and pr36) were designed to form base pairs with both the stem and arm regions, whereas the other two were designed to bind only to the stem (pr11) or only to the arm (pr25) region (Figure 1a). Among the five candidates, two (pr36 and pr27) showed strong activation of the RNA switch, consistent with their greater extent of base pairing. This activation was also confirmed by measurements reported in Figures 1–3.

**Figure 2:**
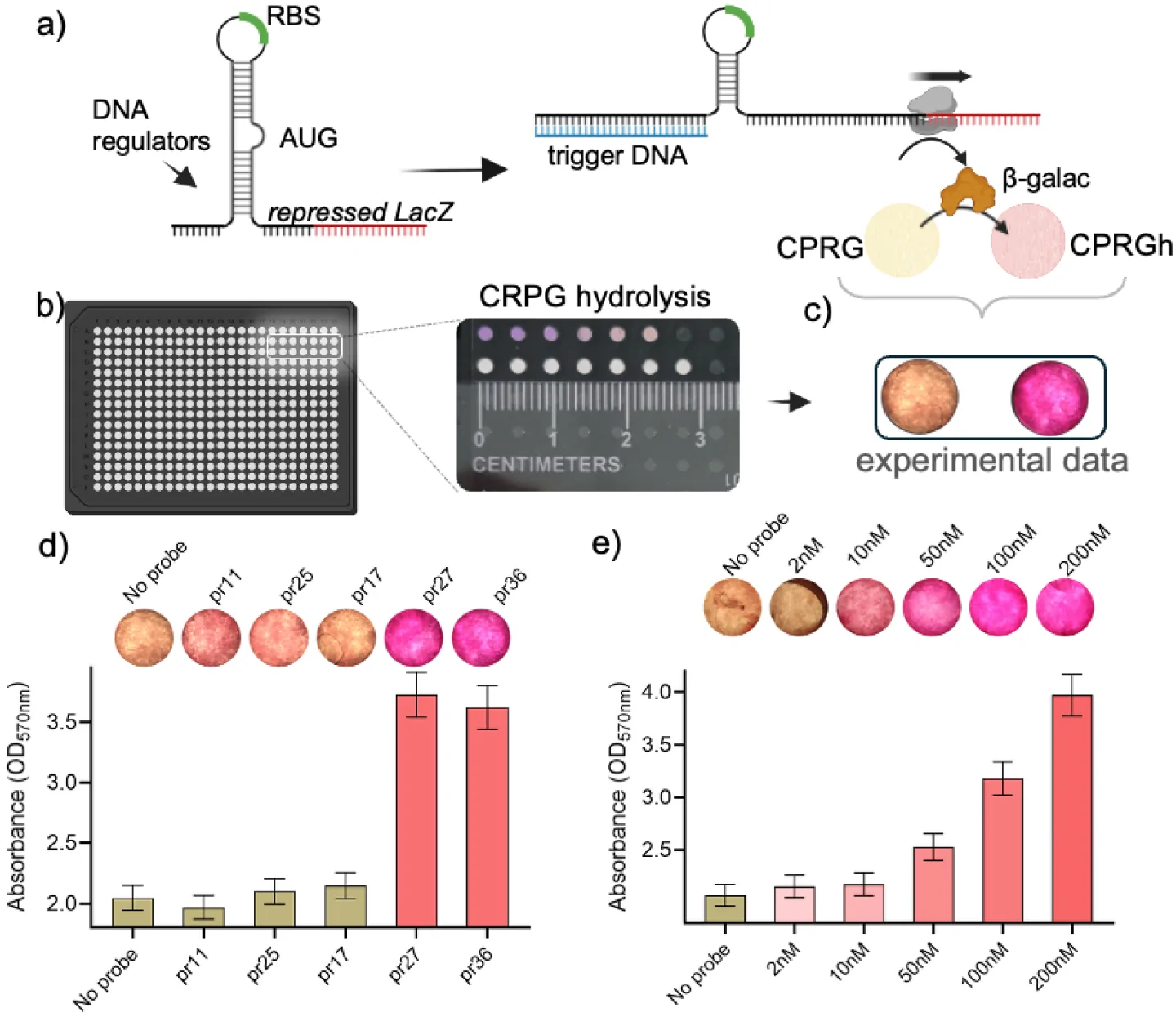
Schematic and experimental workflow for RNA switch activation measured using a colorimetric indicator (CPRG) on paper substrates. (a) Schematic illustration of RNA switch activation with DNA regulators (pr11, pr25, pr17, pr27, and pr36). The enzyme β-galactosidase catalyzes the hydrolysis of CPRG (yellow substrate) into CPRGh (red-purple product) on 2-mm BSA-treated filter paper discs. (b) Paper substrates placed in dark 384-well plates and imaged using a smartphone camera. The lower yellow-colored row shows paper discs without RNA switch activation (absence of the activating pr27 DNA regulator), while the upper red-purple-colored row shows activation in the presence of pr27. (c) Microscopy images of paper discs following CPRG-to-CPRGh conversion under conditions where the riboswitch is either activated (with pr27) or not activated (without pr27). (d) Absorbance measurements at 570 nm collected directly from the paper discs, showing a red color transition for pr27 and pr36, consistent with the anticipated RNA switch activation by these regulators. (e) Concentration-dependent color transition observed on paper discs with increasing levels of the DNA regulator pr27. Readings were collected in 384-well plates at 2 h time points. Experiments were performed in triplicate, and data are presented as mean ± SD.

**Figure 3:**
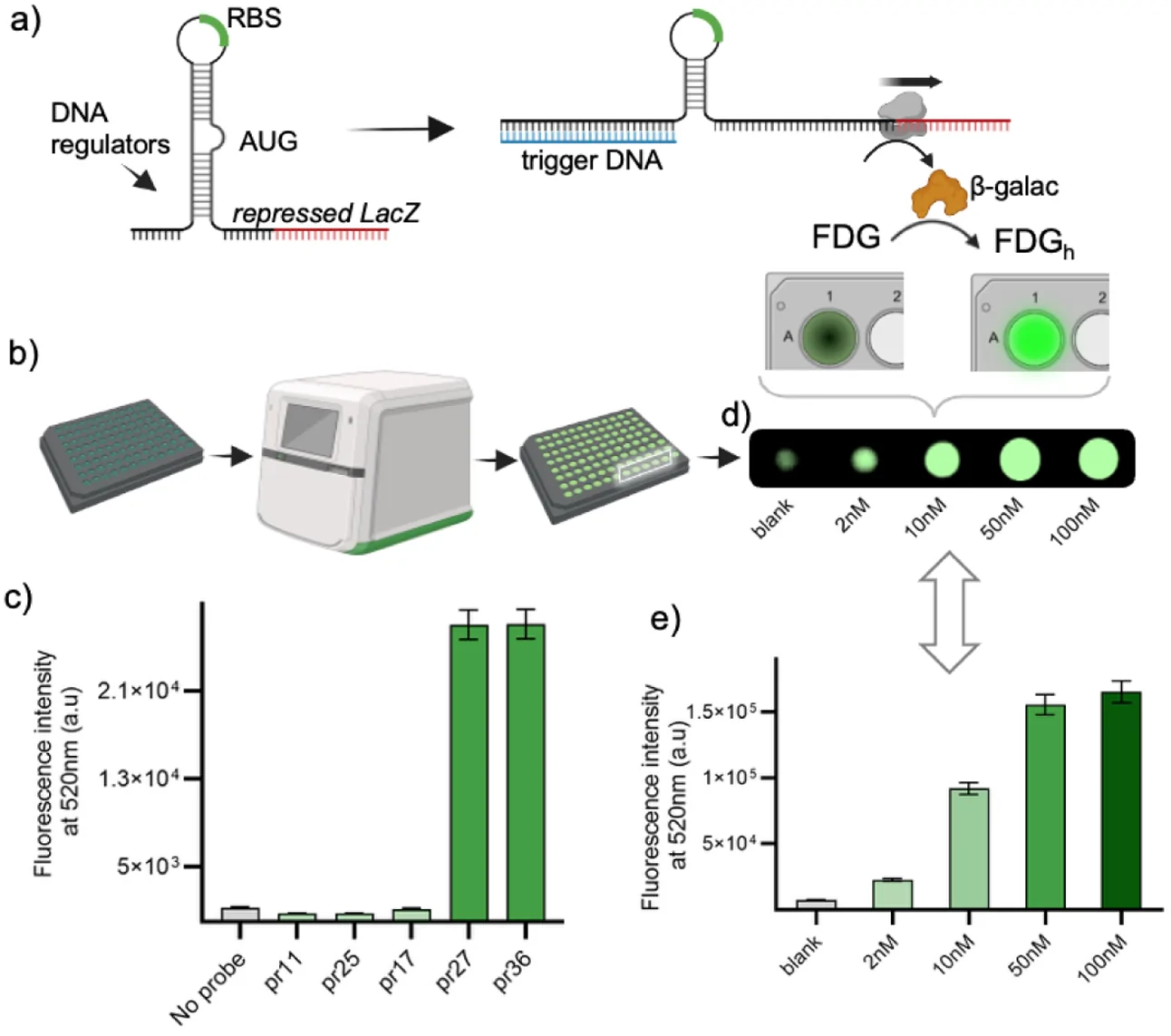
Schematic and experimental workflow for RNA switch activation measured using a fluorogenic indicator (FDG). (a) Schematic illustration of RNA switch activation with DNA regulators. The activating regulators (pr27 and pr36) bind to the RNA switch, enabling expression of the *LacZ* gene and subsequent translation of β-galactosidase. The enzyme catalyzes the hydrolysis of non-fluorescent FDG into the fluorescent product FDGh. (b) Experimental mixtures were placed in 384-well black plates and analyzed using a benchtop spectrofluorometric plate reader. (c) Fluorescence measurements (λemm ≈ 520 nm) show increased signal for pr27 and pr36, consistent with RNA switch activation, whereas other DNA regulators and the no-regulator negative control remain at background levels. (d) Representative, non-quantitative fluorescence images of microplate wells acquired using a gel imaging system, and (e) quantitative fluorescence readings demonstrating a concentration-dependent increase in signal with increasing levels of the DNA regulator pr27. Readings were collected in 384-well plates at 2 h time points. Experiments were performed in triplicate, and data are presented as mean ± SD.

In this design, the DNA regulator (pr36 or pr27) binds to both the stem and arm regions of the RNA switch, destabilizing the folded structure and activating the switch. This exposes the ribosome-binding site and initiates *in vitro* translation. The synthesized β-galactosidase then hydrolyzes lactose in the reaction mixture, generating glucose and galactose.^41^ The resulting glucose was quantified using a handheld glucometer. The β-galactosidase-catalyzed glucose generation, although relatively slow compared with the fluorescence and colorimetric methods reported in Figures 2–3, produced a distinguishable signal after 4 h and a remarkable ∼100-fold increase in the glucometer reading after 24 h with both the pr27 and pr36 activators (Figure 1b). While both the pr27 and pr36 activators resulted in a dramatic signal increase, such behavior was not observed with the control DNA activators (pr11, pr25, and pr17) or the no-activator condition. This can be further visualized in a heatmap, which shows a significant difference in signal output between the pr27 and pr36 activators and the other conditions (Figure 1c).

To confirm that the glucometer signal originated from the cell-free expression system and that no other artifacts arose from the glucometer device itself, two additional reporters were used: one colorimetric and one fluorescent. Chlorophenol red-β-D-galactopyranoside (CPRG) is a yellow substrate that is cleaved by β-galactosidase to produce a red-violet product.^42^ Fluorescein di-β-D-galactopyranoside (FDG), on the other hand, is a fluorogenic substrate that, upon enzymatic cleavage, releases a small fluorescent compound, generating a strong fluorescent emission at 520 nm.^43^ These orthogonal readouts were used to further validate that activation of the RNA switch led to functional enzyme production while also providing alternative approaches when visual and fluorescent detection modalities are preferred.

The experiments were initially performed using pr27 (trigger DNA) in a small 384-well plate with 2-mm BSA-treated filter paper discs loaded with CPRG (Figure 2a–c). Figure 2b shows the 384-well plate and the paper discs in the wells used in this study, along with the observed color transitions. The lower row represents the control samples without pr27, while the upper row shows samples treated with pr27 in images captured with a handheld smartphone camera. A clear color transition on the paper discs was observed when imaged using bright-field microscopy with an attached camera (Figure 2c). The study was then expanded to include all five DNA regulator candidates. As shown in Figure 2d, activation of the RNA switch by pr27 and pr36 resulted in a distinct yellow-to-red color change (CPRG to CPRG^+^) on the paper supports. The remaining three DNA regulators, used as control strands, did not exhibit such a color transition.

The colorimetric assay was further evaluated by depositing varying concentrations of CPRG on paper (Figure S1), in both the presence and absence of pr27. Only samples treated with the activating DNA regulator (pr27) exhibited a clear color transition, with the signal intensity depending on the CPRG concentration. When the CPRG concentration was held constant, increasing the pr27 concentration led to a corresponding increase in color intensity, further confirming controlled RNA switch activation (Figure 2e). Overall, the colorimetric results presented in Figure 2 are in good agreement with the glucometer-based measurements shown in Figure 1.

To further validate *in vitro* activation of the RNA switch, we investigated an alternative measurement modality using a fluorescent indicator. As described above, β-galactosidase translation results in an increase in fluorescence upon cleavage of the fluorogenic substrate, FDG (Figure 3a). These studies were performed in the same 384-well plate format but in solution, with fluorescence measured with a plate reader (Figure 3b). DNA regulators pr27 and pr36, designed to activate the RNA switch and induce β-galactosidase translation, produced a significant increase in the fluorescence signal. All five DNA regulators were evaluated and, consistent with the glucometer-based readings and paper-based colorimetric results, pr27 and pr36 demonstrated the strongest activation (Figure 3c and Figure S2a-b).

As subsequent CRISPR-based studies were performed using pr27 (trigger DNA), we further examined its concentration-dependent activity. Increasing pr27 concentration resulted in a corresponding increase in fluorescence intensity, confirming DNA regulator-dependent activation of the RNA switch (Figure 3d,e and Figure S2e-f). In addition, varying FDG concentration led to a substrate-dependent increase in fluorescence emission at 520 nm (Figure 2c,d).

Overall, results obtained using these two orthogonal indicators (CPRG and FDG) demonstrate that RNA switch activation can be qualitatively and quantitatively monitored both visually on paper and via fluorescence with a benchtop plate reader, in strong agreement with glucometer readouts.

### Pairing the RNA Switch with Cas12a for Detection of a Conserved Genomic Region of a Pathogen

Activating the RNA switch using sequence-specific DNA regulators and measuring the resulting activity with a glucometer, supported by two orthogonal measurement modalities, is highly interesting from a scientific standpoint. However, adapting this construct to a practical application would significantly enhance its translational impact. The CRISPR–Cas12a system provides this capability by functioning upstream in the reaction, where it can regulate the concentration of the DNA regulator substrate, in this case, pr27.^12,17,18,37^ The design and synthesis of crRNAs for the Cas12a–crRNA complex are straightforward using online design tools (e.g., the OligoAnalyzer tool from Integrated DNA Technologies), and can be readily prepared using automated oligonucleotide synthesis. Upon binding to its target sequence via the crRNA, Cas12a becomes activated and exhibits nonspecific single-stranded DNA (ssDNA) cleavage activity.^14,15,44,45^

In this system, activated Cas12a cleaves the trigger DNA (pr27), thereby modulating its availability for downstream RNA switch activation (Figure 4a,b). Target recognition activates the Cas12a–crRNA complex, leading to degradation of the trigger DNA. The degraded trigger is unable to activate the RNA switch and, consequently, cannot initiate translation of β-galactosidase, resulting in no signal generation (Figure 4a). In contrast, in the absence of the target (or in the presence of a non-target), the Cas12a–crRNA complex remains inactive and does not cleave pr27. The intact trigger DNA then activates the RNA switch, enabling translation of β-galactosidase and producing a strong signal across all detection modalities (Figure 4b).

**Figure 4:**
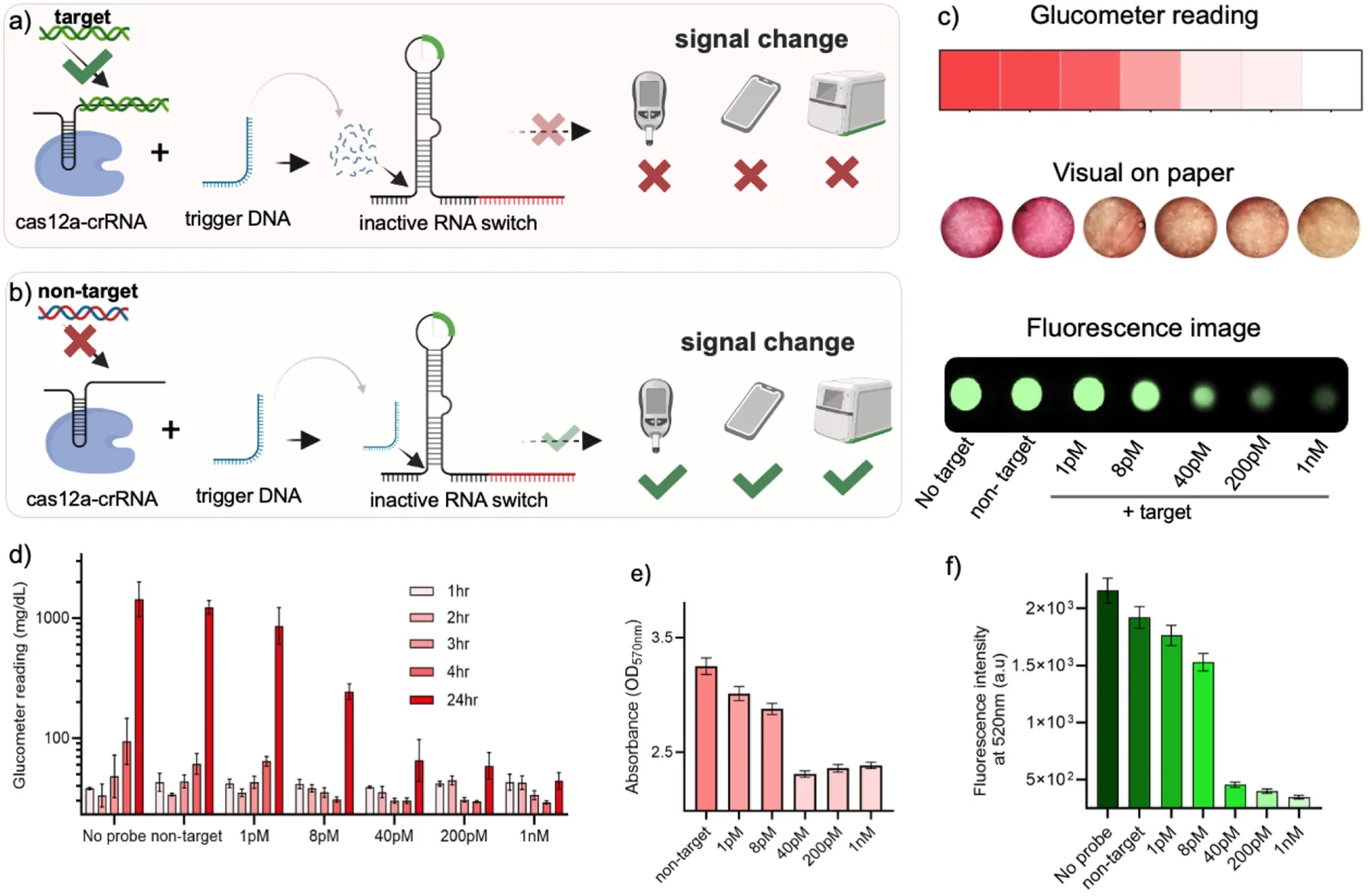
Schematic illustration of RNA switch-CRISPR workflow and experimental results. (a) Target recognition activates the Cas12a–crRNA complex, leading to cleavage of the DNA regulator (pr27). The degraded regulator is unable to activate the RNA switch and subsequently can’t initiate translation of β-galactosidase, resulting in no signal generation. (b) In the absence of target (or in the presence of a nontarget), the Cas12a–crRNA complex remains inactive and does not cleave the DNA regulator (pr27). The intact regulator activates the RNA switch, enabling translation of β-galactosidase and producing an increased signal across all detection modalities. (c) Experimental results across all three detection modalities show that increasing target concentration leads to a progressive decrease in signal (glucometer readings at 24 h, colorimetric response at 2 h, and fluorescence at 2 h). In contrast, no-target and non-target controls exhibit strong signals, including high glucometer readings, a dominant yellow-to-red color transition, and increased fluorescence. (d) Quantitative glucometer readings measured at 1, 2, 3, 4, and 24 h. (e) Absorbance measurements at 570 nm on paper discs at 2 h. (f) Fluorescence measurements (λemm ≈ 520 nm) collected at 2 h in 384-well plates. Experiments were performed in triplicate, and data are presented as mean ± SD.

As shown in Figure 4c, in the absence of the target or in the presence of a nontarget genomic region that is not complementary to the crRNA, strong signals are observed, including elevated glucometer readings, a clear color transition on paper, and increased fluorescence in imaging assays. As the target concentration increases, the signal decreases proportionally, as evidenced by lower glucometer readings, diminished red color intensity (retention of yellow) on paper supports, and reduced fluorescence. Glucometer readings are in the thousands under no-target or nontarget conditions but begin to decrease at target concentrations as low as 1 pM (Figure 4c). This trend is consistently observed across all detection platforms, including paper-based colorimetric assays (CPRG) and fluorescence measurements using a plate reader. Quantitative analysis of the signals was performed using glucometer readings (Figure 4d), absorbance at 570 nm (Figure 4e), and fluorescence intensity at 520 nm (Figure 4f). Although this system operates through a turn-off mechanism, its robust, concentration-dependent performance across all three signaling modalities highlights its potential as a versatile detection platform.

### Programming the RNA Switch-CRISPR Cascade for Detection of Five Bacterial Genomes

To demonstrate that the RNA Switch-CRISPR platform can be selectively reprogrammed for different targets, five distinct Cas12a–crRNA constructs were designed to recognize a conserved genomic region from one of five bacterial pathogens (Figure 5a). Targetable regions within each genome were selected for *Listeria monocytogenes* (*Listeria*), Shiga toxin-producing *E. coli* (*STEC),* *Campylobacter jejuni* (*C. jejuni*), *Salmonella typhi* (*S. typhi*), and *Salmonella enterica* *(* *S. enterica*), based on the presence of a canonical TTTN protospacer adjacent motif (PAM) and relatively high GC content, using sequence analysis with the NCBI BLAST tool.

**Figure 5:**
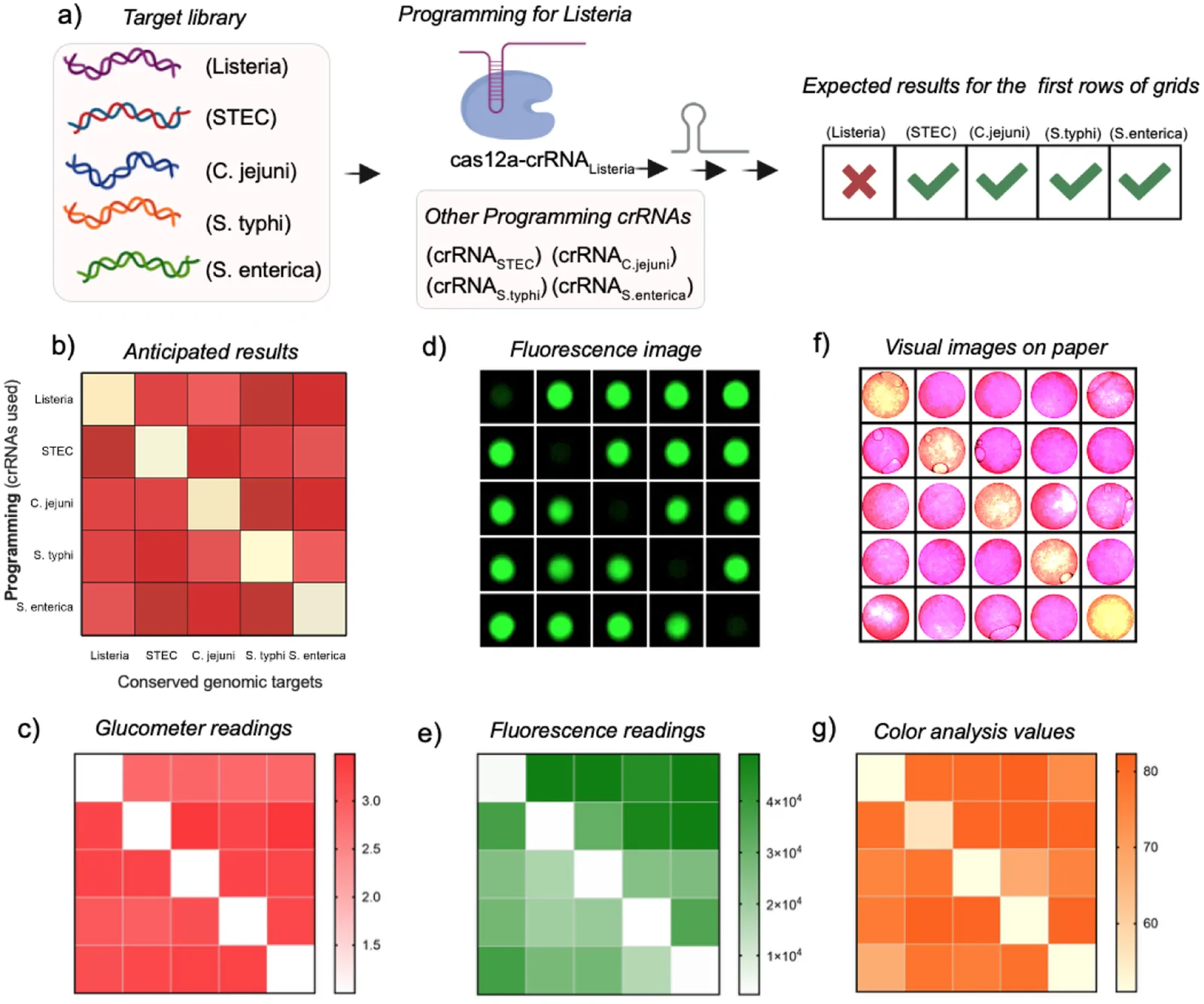
Programmable RNA switch-CRISPR workflow for the detection of conserved genomic regions from multiple bacterial pathogens. (a) Schematic illustration of programming the RNA switch-CRISPR assay to target conserved genomic regions identified from five different bacterial pathogens. Using the *Listeria* target as an example, no change in signal is expected only in the presence of the correct target, whereas non-target (reference) samples are anticipated to produce strong signals across all detection modalities. (b) Design of a 5 × 5 grid assay, where rows correspond to different crRNAs and columns correspond to target inputs. (c) Schematic heatmap illustrating the anticipated response across all three detection modalities. (d) Heatmap of glucometer readings recorded for all 25 conditions at 24 h post-reaction. (e) Representative fluorescence image of the 5 × 5 grid acquired 2 h post-reaction, showing background-level fluorescence for correct target-crRNA pairs and strong fluorescence for non-target combinations, consistent with the expected results. (f) Quantitative fluorescence measurements (λemm ≈ 520 nm) presented as a heatmap. (g) Colorimetric response imaged using a camera-attached microscope , showing strong color transition in non-target controls and significantly reduced signal in target-containing wells. Image quantification was performed using RGB analysis, and results are expressed as red-to-green intensity ratios. All three detection modalities across all 25 conditions show complete agreement with the anticipated outcomes (100% accuracy). Experiments were performed in triplicate, and data are presented as mean ± SD.

A comprehensive 5 × 5 grid experiment was performed to evaluate specificity, where each row corresponds to a system programmed for a specific pathogenic genome, and each column represents exposure to one of the five genomic targets (Figure 5a). In this design, a no change in signal is expected only when the programmed crRNA matches the corresponding genomic sequence, while all nontarget conditions (reference or control wells) should yield strong signal outputs. For example, in the first row (programmed for *Listeria*), a no change in signal is expected in the first well (Figure 5a and first row in 5b) whereas all other genomic inputs are expected to produce high signals across glucometer, colorimetric, and fluorescence readouts. The remaining rows, programmed for STEC, *C. jejuni*, *S. typhi*, and *S. enterica* are expected to exhibit equivalent behavior, as summarized in the grid heatmap (Figure 5b).

Experiments were performed with the RNA switch-CRISPR assay for each experimental and control scenario, and glucometer readings were collected at 24-, 48-, and 72-hour time points. Over time, the glucometer signal increased in non-target conditions several hundredfold, while the matched target conditions consistently showed minimal change, maintaining low signal output (Figure S3a and Figure 5c). Fluorescence measurements were obtained by using two complementary approaches. First, qualitative fluorescence imaging was performed using a benchtop digital imaging platform, revealing clear differences in fluorescent images between target and non-target conditions (Figure 5d). For quantitative analysis, fluorescence intensities at 520 nm were measured and recorded using a spectrophotometric microplate reader, and the resulting quantitative data (Figure S3c) were used to generate the fluorescence heatmap in Figure 5e.

Colorimetric responses on paper substrates were also captured using a TS100 microscope equipped with a digital camera. Red-purple coloration was observed in control wells, whereas experimental wells predominantly retained a yellow color on paper supports. Because of variability introduced by paper folding and surface heterogeneity, direct absorbance measurements at 570 nm were unreliable. Instead, images were quantitatively analyzed using MATLAB-based processing following conversion from RGB to CIE L*a*b* color space, with a focus on the a* channel (red-to-green color axis).^40^ Approximately 17,000 pixels were analyzed per image, enabling pixel-level intensity mapping (Figure S4). These values were then averaged to construct the heatmap presented in Figure 5g and Figure S3b.

This comprehensive study demonstrates remarkable agreement with the expected outcomes illustrated in the heatmap (Figure 5b) across all 25 conditions and all three detection modalities, achieving 100% accuracy (Figure 5c, e, and g). Quantitative analysis using glucometer readings, fluorescence intensity, and image-based colorimetric metrics further confirms the high selectivity and robustness of the programmable RNA switch-CRISPR assay (Figure 5).

### Full-Genome Detection with Positive and Negative Controls and Sensitivity Evaluation

To this end, we evaluated the assay using conserved genomic regions identified from the NCBI genome database and refined target selection through sequence analysis with the NCBI BLAST tool. To demonstrate the applicability of the platform to real-world samples, we extended the study to whole genomic DNA isolated from *Listeria monocytogenes*. Experiments were performed with multiple positive and negative controls to assess the selectivity, robustness, and sensitivity of the assay. The *Listeria* genome was first subjected to an isothermal amplification step using recombinase polymerase amplification (RPA), which operates at room temperature and enables rapid amplification of target sequences within 30 min, Figure 6a. Prior to RPA, eight primers (four forward and four reverse) were designed using SnapGene to generate amplicons within the target genomic regions, Table S1. Sixteen forward-reverse combinations were tested in the presence and absence of the target genome using a Cas12a F-Q assay.^15^ Although all 16 combinations performed effectively, the F4/R2 primer set was selected for subsequent studies (Figure S7). Different amounts of the resulting amplicons from the full *L. monocytogenes* genome (1.25, 2.5, 5, 10, 50, and 100 copies/μL) originating from RPA product stocks were then introduced into the RNA-switch-CRISPR assay programmed specifically for *L. monocytogenes* detection. The results showed that as few as 1.25 copies/μL of the full *L. monocytogenes* genome, used for preparing RPA amplicons, can be detected using a handheld glucometer (Figure 6a,b). In our previous work, utilizing a CRISPR-RPA system alone demonstrated detection of whole genomes at concentrations as low as 40 copies.^37^ In the present study, our RNA-switch-CRISPR-based detection system coupled with RPA enables detection down to a single copy, suggesting that the additional RNA-switch step enhances the overall analytical sensitivity of the platform.

**Figure 6:**
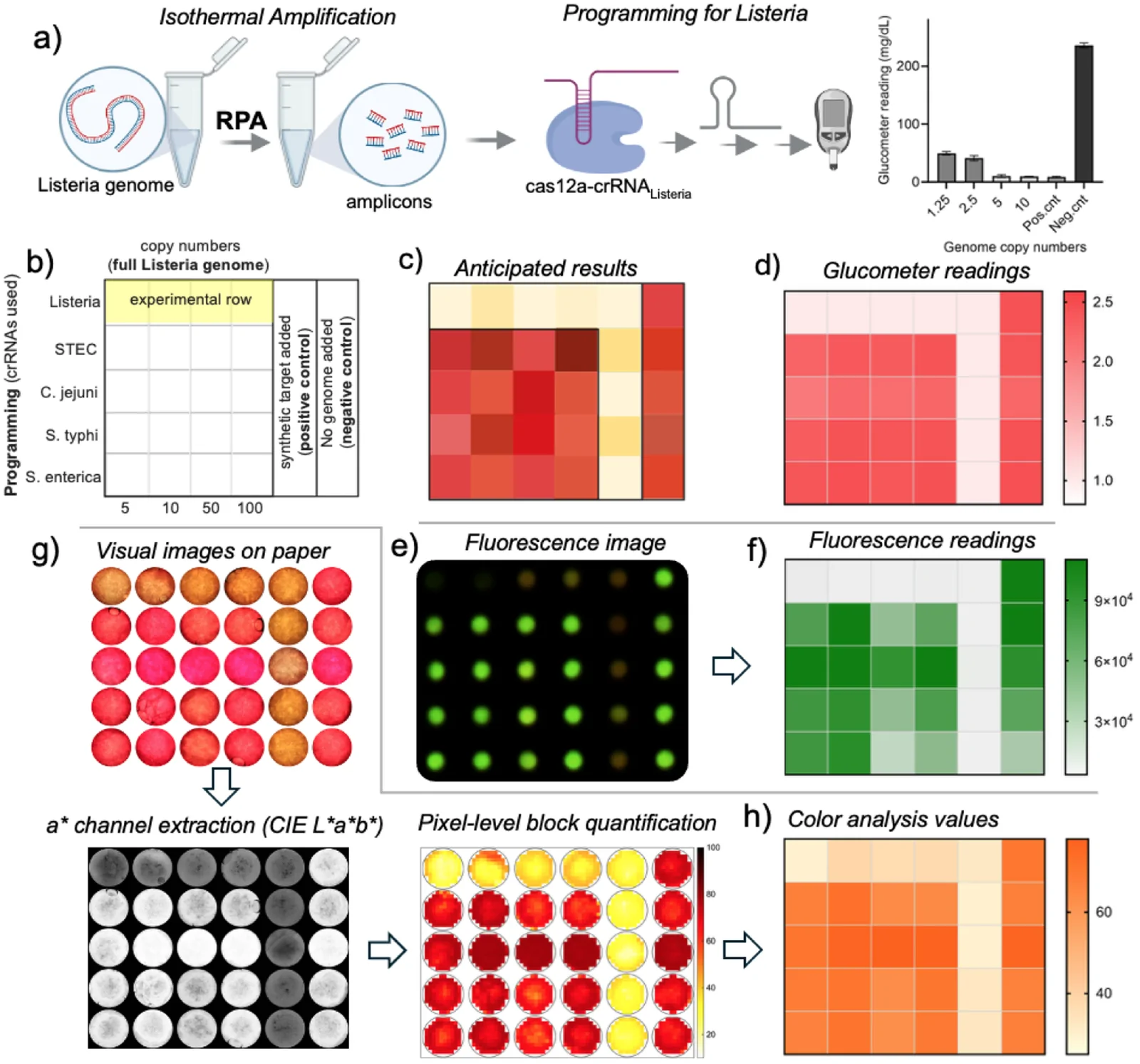
Detection of full bacterial genome using RPA-assisted RNA switch-CRISPR with three detection modalities. (a) Schematic illustration of the RNA switch-CRISPR assay programmed to detect the full *Listeria* genome following upstream recombinase polymerase amplification (RPA), alongside corresponding glucometer results presented as bar graphs (24 h post-reaction). (b) A comprehensive 6 × 5 grid assay design evaluating specificity and sensitivity, with various control studies. The first row represents reactions performed with the crRNA targeting *Listeria* across varying concentrations of RPA-generated amplicons. The remaining rows correspond to control (reference) crRNAs tested against the same amplicons. The final column represents the negative control (no genome added to the RPA reaction), whereas the second-to-last column includes reactions using synthetic amplicons matched to each crRNA target. (c) Schematic heatmap illustrating the anticipated response across all three detection modalities. (d) Heatmap of glucometer readings recorded for all 30 conditions at 24 h post-reaction. (e) Representative fluorescence image of the 6 × 5 grid acquired 2 h post-reaction, showing background-level fluorescence for correct target–crRNA combinations (i.e., successful detection of *Listeria*). (f) Quantitative fluorescence measurements (λemm ≈ 520 nm) presented as a heatmap. ((g) Colorimetric response imaged under camera-attached microscopy, showing strong color transition in non-target controls and significantly reduced signal in target-containing wells. Quantitative image analysis was performed on the RGB images following conversion to CIE L*a*b* color space, with analysis restricted to the a* channel (red-to-green color axis transformation). Circular assay regions (2 mm diameter; ∼17,000 pixels) were extracted, and block-wise mean a* values were computed to generate the spatial heatmap within assay regions. (h) Overall block-wise mean a* colorimetric signal. All positive (no signal change) and negative (signal-generating control) conditions performed as expected. All three detection modalities across all 30 conditions show complete agreement with the anticipated outcomes (100% accuracy). Experiments were performed in triplicate, and data are presented as mean ± SD.

To evaluate selectivity, crRNAs targeting four other bacterial genomes—STEC, *C. jejuni*, *S. typhi*, and *S. enterica*, were used as reference wells (rows 2–5 (Figure 6b). As a negative control, reactions were performed in the absence of *the Listeria* genome but included the RPA step (Figure 6b, last column). As positive controls, synthetic amplicons for each crRNA were introduced into the assay to demonstrate that the assay is in fact functional (Figure 6b, second-to-last column). In total, 30 different experimental combinations were tested (Figure 6b).

As illustrated in the heatmap (Figure 6c), a reverse “L”-shaped pattern is expected across all measurement modalities. The results confirm this behavior: strong glucometer signals are observed in all reference wells in which the crRNAs are programmed against non-*Listeria* genomic targets (rows 2–5), whereas low glucometer signals are consistently observed across all tested amounts of the full *Listeria* genome (experimental row 1). The negative and positive control columns exhibit responses consistent with the anticipated outcomes. The heatmap shown in Figure 6d is constructed from the glucometer readings presented in Figure S5a.

In parallel, the assay was repeated using the fluorogenic substrate FDG, and fluorescence across the array was recorded by qualitative imaging with a benchtop digital imaging platform, revealing clear differences in the fluorescent images (Figure 6e), and was quantified via fluorescence intensity measurements at 520 nm using a spectrophotometer (Figure S5c). The resulting heatmap (Figure 6f) shows strong agreement with the glucometer-based results in Figure 6d.

Finally, the study was extended to the colorimetric format on 2 mm-sized paper chips using CPRG as an indicator. Color transitions on the paper substrate were captured using a camera-attached microscope and quantitatively analyzed using RGB-derived CIE L*a*b* color-space based-image processing (Figure 6g), as described above. Averaged a* intensity values (Figure S5b) were used to construct a heatmap (Figure 6h), which shows strong agreement with the glucometer and fluorometric results in Figure 6d and f. These results demonstrate that all three detection modalities produce consistent outputs that align with expected trends.

Although the glucometer-based measurements alone provide a clear and quantitative readout, the inclusion of orthogonal colorimetric and fluorometric detection modalities and extensive control conditions further underscores the robustness and reliability of the assay. Notably, low copy numbers of the complete pathogenic genome could be detected using a low-cost glucometer, highlighting the strong potential of this platform for point-of-care diagnostic applications. The parameters, advantages, and limitations of each detection modality are summarized in Table 2. The glucometer-based approach provides a robust and quantitative readout using a portable device, although it requires a longer assay time. In contrast, fluorescence provides a faster quantitative readout but requires a fluorescence plate reader and is subject to fluorophore stability constraints, whereas the colorimetric approach enables instrument-free visual detection, although quantitative analysis requires additional image or absorbance measurements and may be affected by variation in color interpretation.

**Table 2:** Summary of Three Detection Modalities

	Feature of Glucometer-based	Fluorescence	Visual Colorimetric
**Substrate**	Lactose	FDG	CPRG
**Quantitative**	Yes	Yes	Semi-quantitative
**Sensitivity** (without RPA)	1 pM	40 pM	40 pM
**Advantages**	Robustness	Fast	Instrument-free
**Instrumentation**	Handheld glucometer	Spectrophotometer	Digital camera
**Limitations**	Slow	Fluorophore stability	Color deviation

## Conclusion

Here, we have developed a programmable genome detection platform through cell-free *in vitro* translation of β-galactosidase that integrates an RNA switch with CRISPR–Cas12a. The assay enables signal generation across three distinct output modalities. By coupling target genome recognition through CRISPR–Cas12a to β-galactosidase expression via RNA switch activation, this system achieves substantial signal amplification, allowing detection using a low-cost (∼$15) handheld glucometer, alongside fluorescence measurements in solution and colorimetric readouts on 2-mm-sized filter paper supports. The assay was systematically validated through rigorous control experiments and cross-modality comparisons, demonstrating strong agreement between glucometer-based measurements, fluorescence signals, and colorimetric outputs. The platform is readily reconfigurable and enables selective detection of conserved genomic regions across multiple bacterial pathogens without redesigning the core RNA switch architecture. Integration of CRISPR-mediated upstream processing with downstream RNA switch activation establishes a modular and scalable sensing platform, achieving detection of as few as nearly a single copy of the full *Listeria monocytogenes* genome in a 1 μL sample. This design enables programmable targeting across multiple genomes using a unified RNA switch architecture, minimizing variability associated with redesigning distinct RNA switches for each target.

## Supplementary Material



## Data Availability

The data supporting the findings of this study are available within the article and its Supporting Information. The MATLAB code for the colorimetric analysis, along with representative test images provided solely for demonstration and code execution, is available on GitHub at https://github.com/Edem2326/Yigit-lab-CPRG-colorimetric-image-analysis.git. All datasets supporting this colorimetric analysis, including raw and processed images, are available on Zenodo at 10.5281/zenodo.19556047. Additional data are available from the corresponding author upon reasonable request.

## Notes

Oligonucleotide sequences (**Table S1**) and additional data (**Figures S1–S7**) (PDF)
