# Supplementary material for: Programmable Genome Sensing with Visual, Fluorescence, and Glucometer Readings: Cell-Free In Vitro Translation Approach Using RNA Switch-CRISPR
Source: Biochemistry. 2026 Sep 16;65(19):2972–85. doi: 10.1021/acs.biochem.6c00564 (PMC13648225; doi:10.1021/acs.biochem.6c00564)
Supplement: Supplementary file 1 [file bi6c00564_si_001.pdf]

SUPPORTING INFORMATION

Programmable genome sensing with visual, fluorescence and glucometer readings: Cell-free in vitro translation approach using RNA switch-CRISPR

Mahla Lashkari[†,‡], Natalie E. Connell[†], Emmett Hanson[†], Emmanuel E. Adade[†], Ram J. Tharu[†] and Mehmet V. Yigit[*,†,‡]

† Department of Chemistry,
University at Albany, State University of New York,
1400 Washington Avenue, Albany, New York 12222, United States.

‡ The RNA Institute,
University at Albany, State University of New York,
1400 Washington Avenue, Albany, New York 12222, United States.

*Correspondence:

Tel: (1) 518-442-3002

myigit@albany.edu

Table S1. List of oligonucleotide sequences used. For crRNAs, the target recognition region is bolded and underlined. For truncated target sequences, the recognition sites are bolded and underlined, and PAM sequences are highlighted in green. RBS and Start Codon are shown in purple. FP and RP are the Forward Primer and Reverse Primer, respectively.

RNA switch region	5'UUUCGCUCUAUUCUCAUCAGUUUCAUGUCCUGUGUCGGACUUUAGAACAGAGGAGAUAAAGAUGGACACAGGACACAACCUGGCGGCAGCGCAAAAG3'
Probe 36 (pr36)	5-'GACACAGGACATGAAACTGATGAGAATAGAGCGAAA-3'
Probe 27 (pr27)	5'- GACACAGGACATGAAACTGATGAGAAT-3'
Probe 25 (pr25)	5'- TGAAACTGATGAGAATAGAGCGAAA-3'
Probe 17 (pr17)	5'- GACACAGGACATGAAAC -3'
Probe 11 (pr11)	5'- GACACAGGACA -3'
RNA Probe (prR36)	5'- GACACAGGACAUGAAACUGAUGAGAAUAGAGCGAAA-3'
crRNA *Listeria*	5'- UAAUUUCUACUAAGUGUAGAU**<u>CGCUUACGGCAGCAUCAAAAGC</u>**-3'
crRNA STEC	5'- UAAUUUCUACUAAGUGUAGAU**<u>CGAGUACAUUGGCAUCGUGUGG</u>**-3'
crRNA *C. jejuni*	5'-UAAUUUCUACUAAGUGUAGAU**<u>UCGAGCACUUCCAUGACCACCCC</u>**-3'
crRNA *S. typhi*	5'-UAAUUUCUACUAAGUGUAGAU**<u>UCGAGAUCGCCAAUCAGUCC</u>**-3'
crRNA *S. enterica*	5'-UAAUUUCUACUAAGUGUAGAU**<u>CGAAUAACAUCCUCAACUUC</u>**-3'
Listeria target	Target Sequence: 5'-AAAGTAAAAGCT**<u>GCTTTTGATGCTGCCGTAAGCGGAAA</u>**ATCTGTCTCAGG-3'
	Complementary Sequence: 5'- CCTGAGACAGAT**<u>TTTCCGCTTACGGCAGCATCAAAAGC</u>**AGCTTTTACTTT-3'
STEC target	Target Sequence: 5'-TTTTTACCCTGT**<u>CCACACGATGCCAATGTACTCGGAAA</u>**AATATCAAAAGC-3'
	Complementary Sequence: 5'- GCTTTTGATATT**<u>TTTCCGAGTACATTGGCATCGTGTGG</u>**ACAGGGTAAAAA-3'
C. jejuni target	Target Sequence: 5'-AGTTATTGGAA**<u>GGGGTGGTCATGGAAGTGCTCCAGAAA</u>**AGGCAAAAGATC-3'
	Complementary Sequence: 5'- GATCTTTTGCCT**<u>TTTCTGGAGCACTTCCATGACCACCCC</u>**TTCCAATAACT-3'
S. typhimirium target	Target Sequence: 5'-TCGTCGTTA**<u>GGACTGATTGGCGATCTCGATAAA</u>**GTCTCTACAGAGACCGT-3'
	Complementary Sequence: 5'- ACGGTCTCTGTAGAGAC**<u>TTTATCGAGATCGCCAATCAGTCC</u>**TAACGACGA-3'
S. enterica target	Target Sequence: 5'-GGTATCTGCT**<u>GAAGTTGAGGATGTTATTCGCAAA</u>**GGGATCCGTCAGACCT-3'
	Complementary Sequence: 5'-AGGTCTGACGGATCCC**<u>TTTGCGAATAACATCCTCAACTTC</u>**AGCAGATACC-3'
F1 (forward primer 1)	5'-ATATCTCAAGTGTGGCGTATGGCCGTCAAG-3'
F2 (forward primer 2)	5'-CATATATCTCAAGTGTGGCGTATGGCCGTC-3'
F3 (forward primer 3)	5'-CCTGCATATATCTCAAGTGTGGCGTATGGC-3'
F4 (forward primer 4)	5'-TCCTCCTGCATATATCTCAAGTGTGGCGTA-3'
R1 (reverse primer 1)	5'-TTTGTTAGTTCTACATCACCTGAGACAGAT-3'
R2 (reverse primer 2)	5'-TGATATTTGTTAGTTCTACATCACCTGAGA-3'
R3 (reverse primer 3)	5'-GCCGTCGATGATTTGAACTTCATCTTTTGC-3'
R4 (reverse primer 4)	5'-GAGGTTGCCGTCGATGATTTGAACTTCATC-3'
F-Q Probe	5'-56-FAM-TTTTTTTT-3IABkFQ-3'

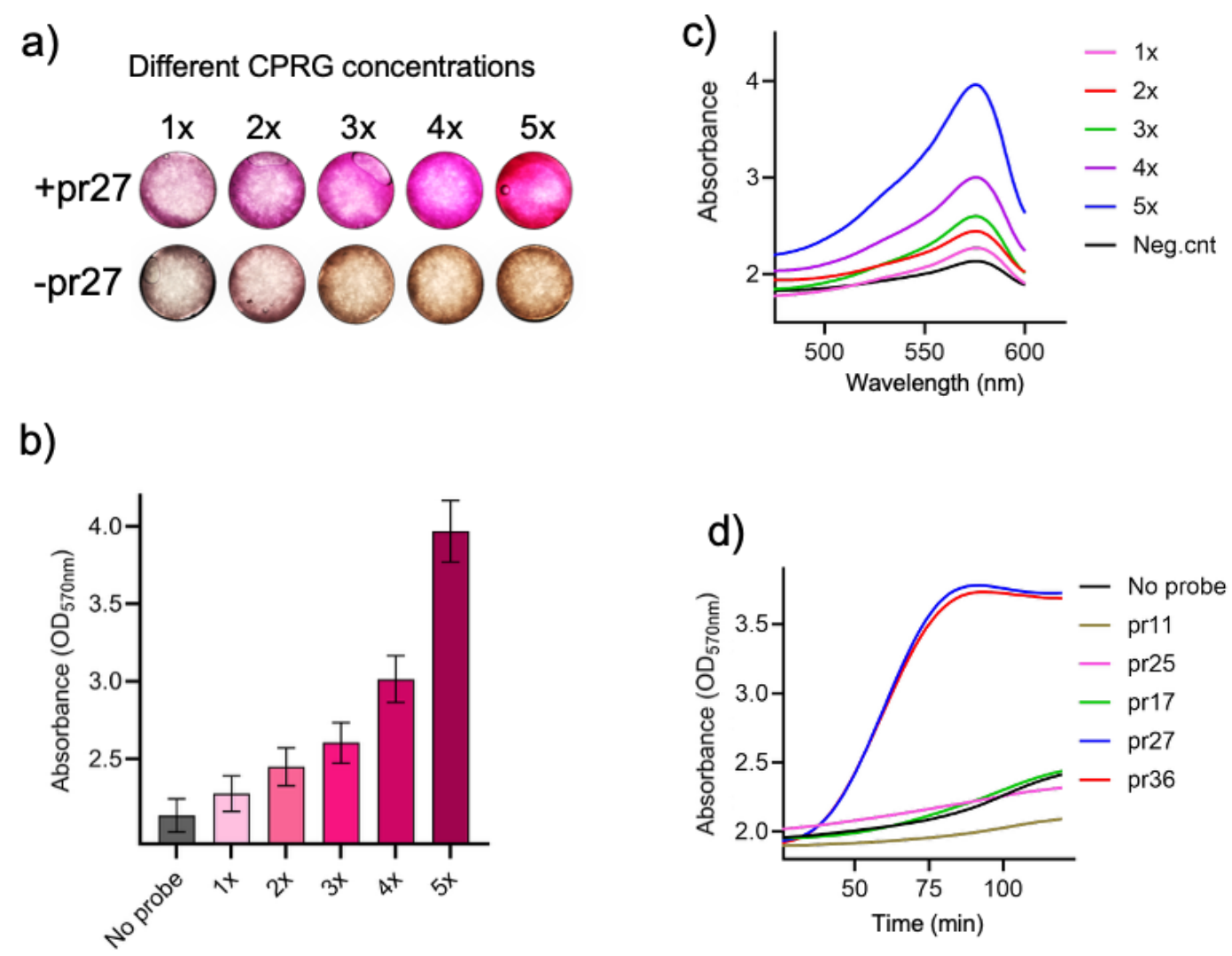

Figure S1. Evaluation of RNA switch activation at varying CPRG concentrations with and without the DNA regulator pr27. (a) Microscopy images of paper discs showing CPRG-to-CPRGh conversion at different CPRG substrate concentrations under conditions where the riboswitch is activated (with pr27) or not activated (without pr27). **(b)** Endpoint absorbance measurements at 570 nm. and **(c)** Absorbance spectra acquired directly from the paper discs at the indicated 2hours time point, **(d)** Time-course absorbance measurements of the colorimetric response at 570 nm over 2 hours, collected in 384-well plates. Experiments were performed in triplicate, and data are presented as mean ± SD.

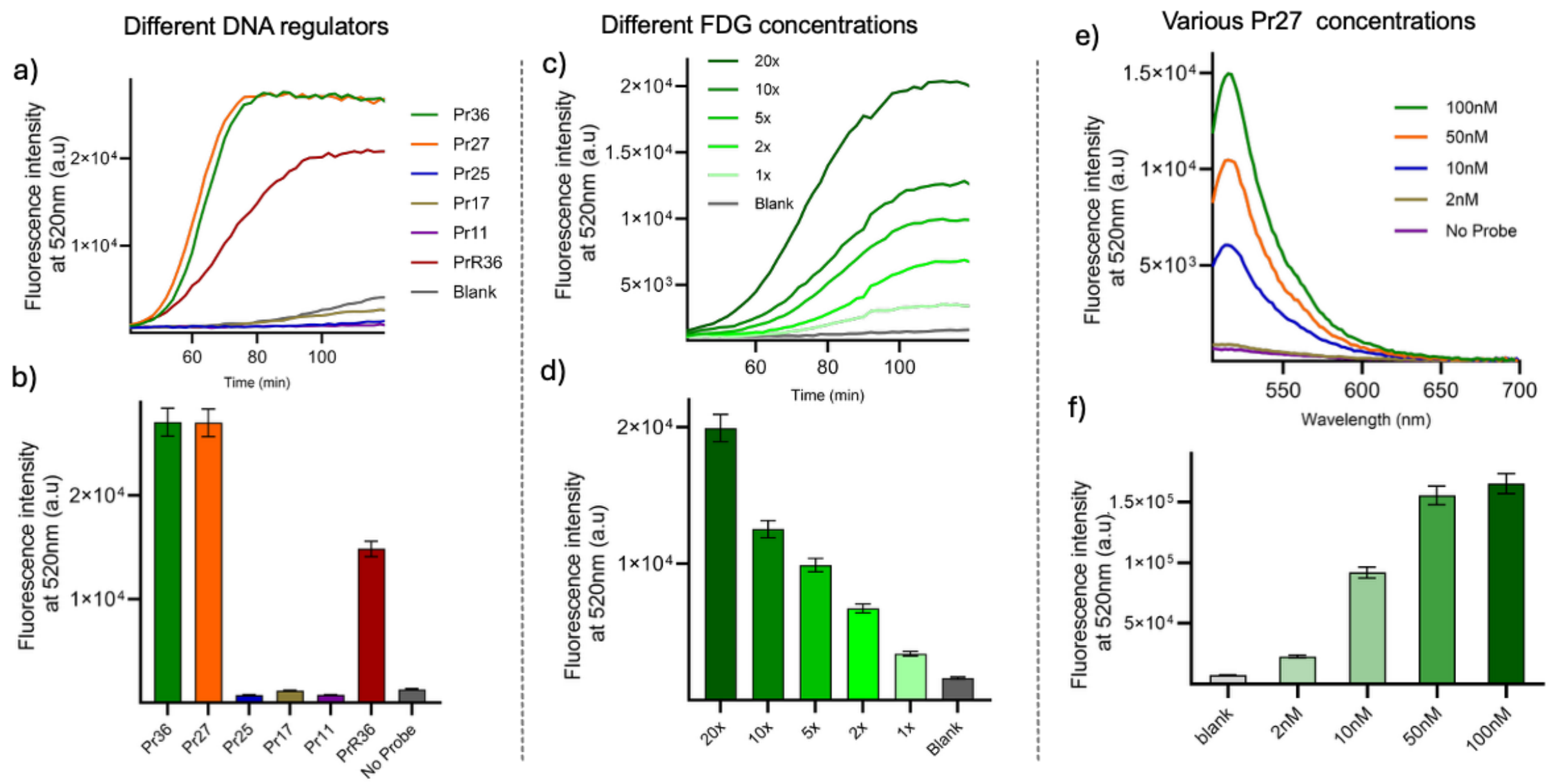

Figure S2. Evaluation of RNA switch activation using FDG with different DNA regulators and varying FDG and pr27 concentrations. (a) Time-course fluorescence measurements (λemm. ≈520 nm) over 2 hours. **(b)** Endpoint fluorescence measurements showing increased signal for pr27 and pr36, consistent with riboswitch activation, whereas other DNA regulators and the no-regulator negative control remain at background levels. prR36 serves as a positive control based on prior reports.[4,5] **(c-d)** Fluorescence response measured across varying FDG concentrations (1x= 0.065) at a fixed concentration of pr27: 50 nM **(c)** time-course measurements over 2 hours and **(d)** corresponding endpoint values. **(e-f)** Fluorescence response measured at a fixed FDG concentration (10x= 0.65) with varying concentrations of pr27: **(e)** time-course measurements and **(f)** corresponding endpoint values collected at 2 hours. All readings were collected in 384-well plates. Experiments were performed in triplicate, and data are presented as mean ± SD.

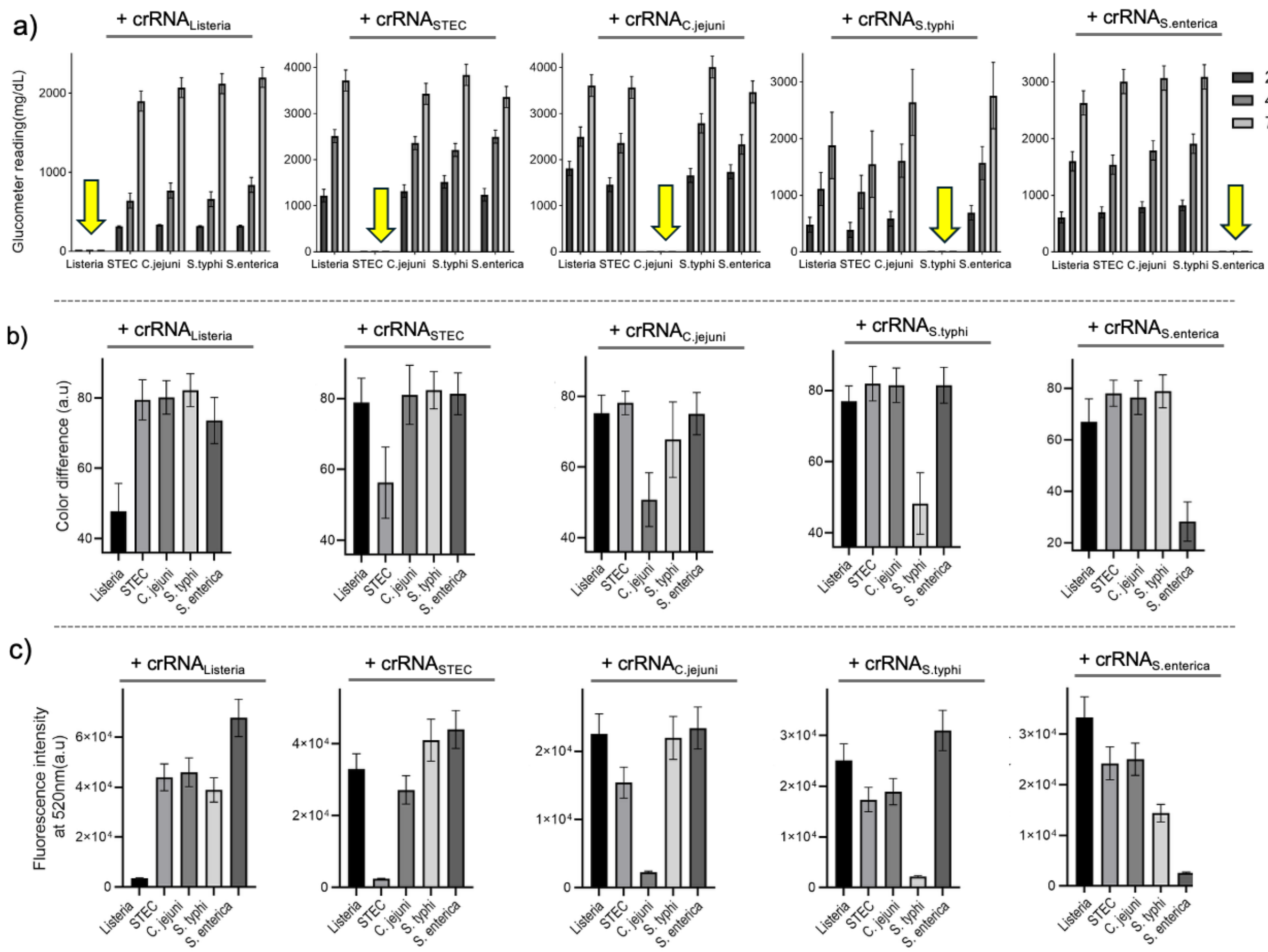

Figure S3. Programmable RNA switch–CRISPR workflow for detection of conserved genomic regions from multiple bacterial pathogens. (a–c) Glucometer readings, colorimetric (RGB) analysis, and fluorescence measurements (λemm. ≈ 520 nm) used for generating 5 × 5 grid heatmap assay reported in **Figure 5**. **(a)** Glucometer readings recorded for all 25 conditions at 24 hours post-reaction. **(b)** Colorimetric response quantified from paper-based images using RGB analysis and expressed as red-to-green intensity ratios. **(c)** Quantitative fluorescence measurements (λemm. ≈ 520 nm) recorded for all 25 conditions at 2 hours post-reaction. Experiments were performed in triplicate, and data are presented as mean ± SD.

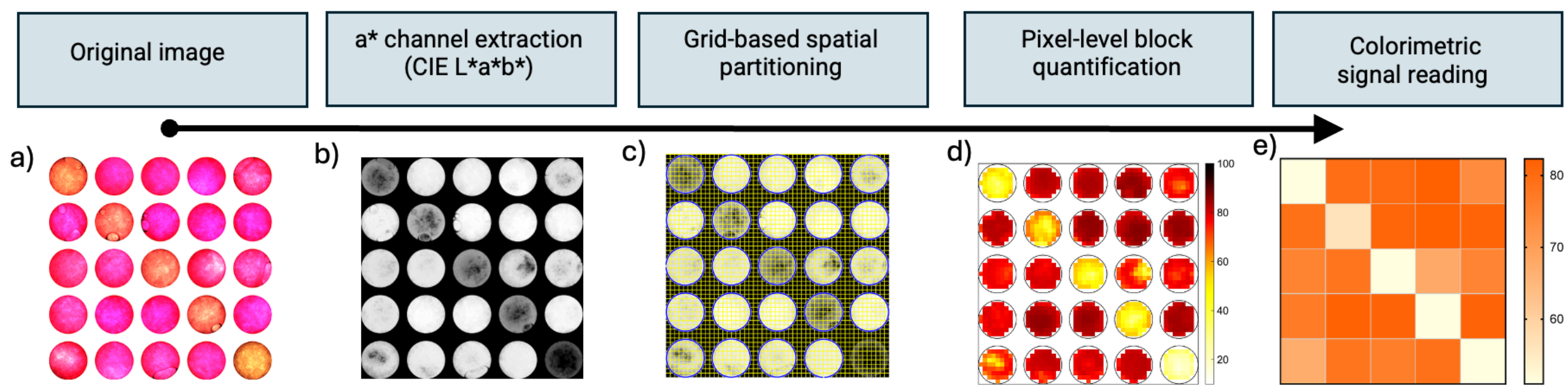

Figure S4. Quantitative analysis of CPRG colorimetric assay images. (a) Composite 5 x 5 grid of individual RGB images from CPRG assays paperclips. Images were adjusted for contrast using identical parameters across all samples **(b)** Conversion of RGB images to CIE L*a*b* color space. This representative image shown corresponds to the a* channel, which represents the red-green color axis. and captures chromogenic changes associated with the CPRG substrate conversion. **(c)** Spatial partitioning of the image into square grid blocks of 15 x 15 pixels across the detected circular assay regions enabling adequate pixel sampling. Only blocks with 95 % area overlap within detected circles were analyzed. **(d)** Block-wise quantification of the mean a* intensity values across retained grid blocks shown as a heatmap corresponding to spatial distribution colorimetric signal in assay regions **(e)** Heatmap visualization of mean a* intensity values, representing the overall block-wise colorimetric signal within the assay regions. Analysis and visualizations were performed in MATLAB **(a-d)** and GraphPad Prism **(e)**.

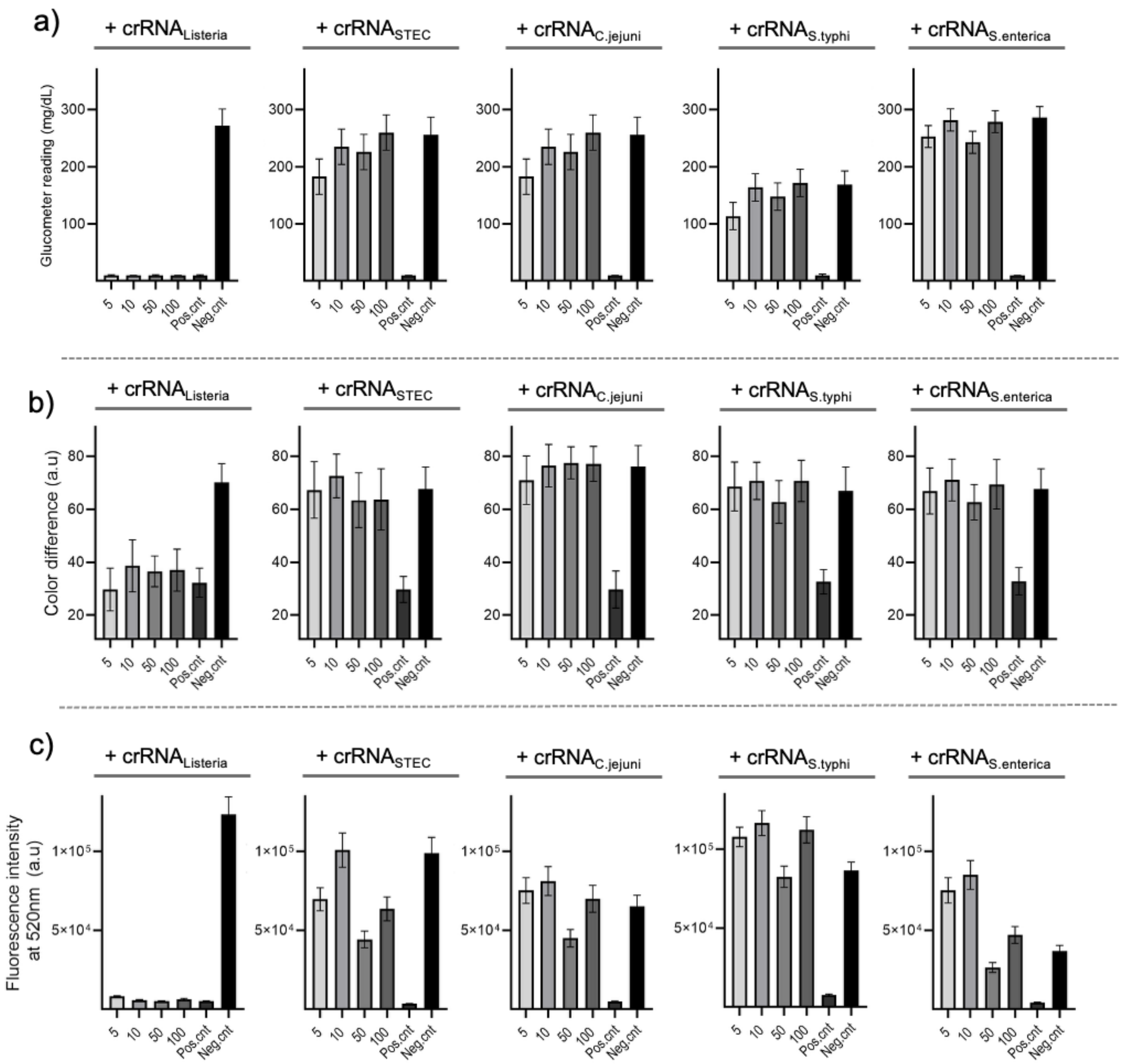

Figure S5. Detection of full bacterial genome using RPA-assisted RNA switch–CRISPR with three detection modalities. (a) (a–c) Glucometer readings, colorimetric (RGB) analysis, and fluorescence measurements (λemm. ≈ 520 nm) used for generating 6 × 5 grid heatmap assay reported in **Figure 4**. **(a)** Glucometer readings recorded for all 30 conditions at 24 hours post-reaction. **(b)** Colorimetric response quantified from paper-based images using RGB analysis and expressed as red-to-green intensity ratios. **(c)** Quantitative fluorescence measurements (λemm. ≈ 520 nm) recorded for all 30 conditions at 2 hours post-reaction. Experiments were performed in triplicate, and data are presented as mean ± SD.

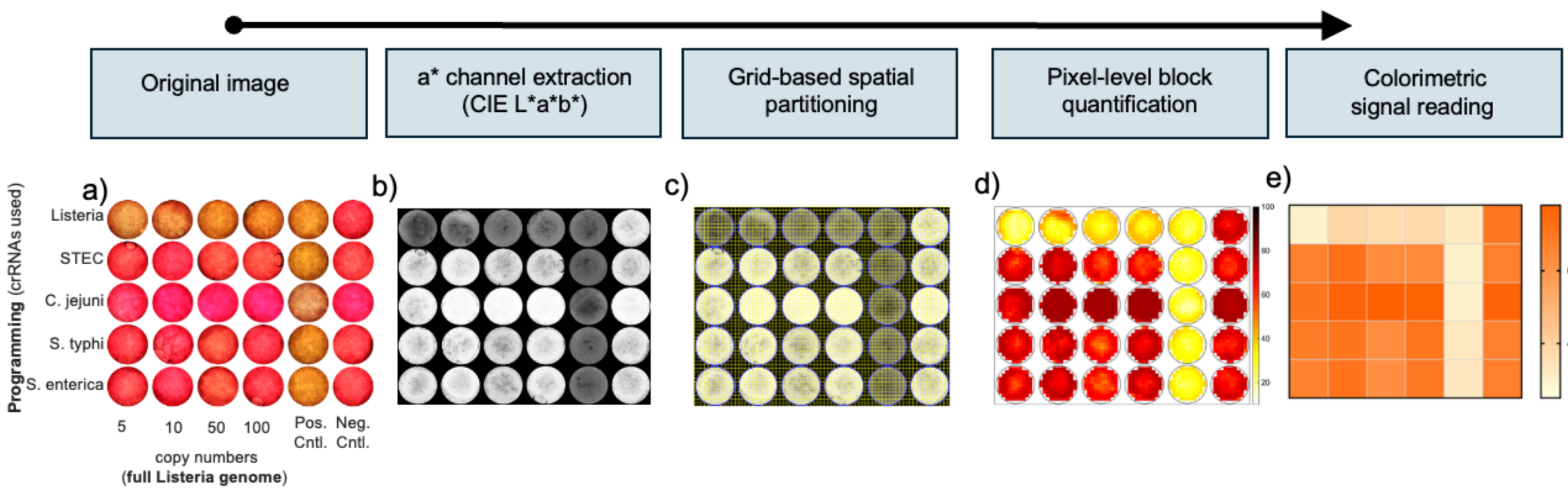

Figure S6. Quantitative analysis of CPRG colorimetric assay images. (a) Composite 5 x 6 grid of individual RGB images from CPRG assays paperclips. Images were adjusted for contrast using identical parameters across all samples **(b)** Conversion of RGB images to CIE L*a*b* color space. This representative image shown corresponds to the a* channel, which represents the red-green color axis. and captures chromogenic changes associated with the CPRG substrate conversion. **(c)** Spatial partitioning of the image into square grid blocks of 10 x 10 pixels across the detected circular assay regions enabling adequate pixel sampling. Only blocks with 95 % area overlap within detected circles were analyzed. **(d)** Block-wise quantification of the mean a* intensity values across retained grid blocks shown as a heatmap corresponding to spatial distribution colorimetric signal in assay regions **(e)** Heatmap visualization of mean a* intensity values, representing the overall block-wise colorimetric signal within the assay regions. Analysis and visualizations were performed in MATLAB (**a-d**) and GraphPad Prism **(e)**.

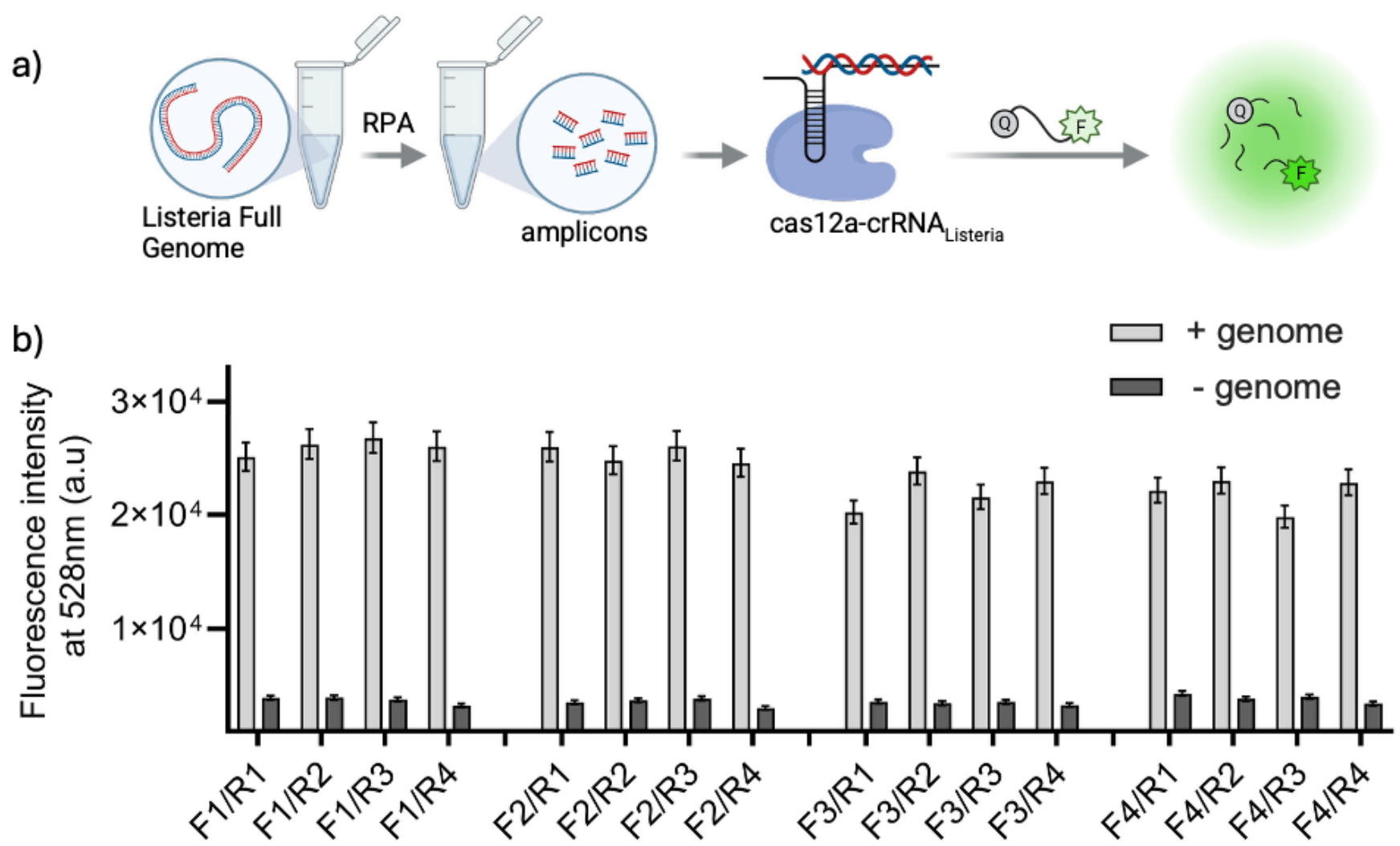

Figure S7. Primer screening for *Listeria monocytogenes* EGD-e. (a) Schematic illustration of primer screening, where each forward and reverse primer pair is evaluated using the *Listeria* genome as a template. **(b)** Screening results for four forward primers and four reverse primers. Light gray bars represent reactions performed in the presence of the genome, while dark gray bars represent no-genome controls. The F4/R2 primer set was selected for all experiments in this study based on its performance. Experiments were performed in triplicate, and data are presented as mean ± SD.
